# Immunologic Characterization and T cell Receptor Repertoires of Expanded Tumor-infiltrating Lymphocytes in Patients with Renal Cell Carcinoma

**DOI:** 10.1158/2767-9764.CRC-22-0514

**Published:** 2023-07-18

**Authors:** Moon Hee Lee, Jason Theodoropoulos, Jani Huuhtanen, Dipabarna Bhattacharya, Petrus Järvinen, Sara Tornberg, Harry Nísen, Tuomas Mirtti, Ilona Uski, Anita Kumari, Karita Peltonen, Arianna Draghi, Marco Donia, Anna Kreutzman, Satu Mustjoki

**Affiliations:** 1 Hematology Research Unit Helsinki, Department of Clinical Chemistry and Hematology, University of Helsinki and Helsinki University Hospital Comprehensive Cancer Center, Helsinki, Finland.; 2 Translational Immunology Research Program, University of Helsinki, Helsinki, Finland.; 3 iCAN Digital Precision Cancer Medicine Flagship, University of Helsinki, Helsinki, Finland.; 4 Department of Computer Science, Aalto University, Espoo, Finland.; 5 Abdominal Center, Urology, Helsinki University and Helsinki University Hospital, Helsinki, Finland.; 6 Department of Pathology, HUS Diagnostic Center, Helsinki University Hospital, Helsinki, Finland.; 7 Research Program in Systems Oncology, Faculty of Medicine, University of Helsinki, Helsinki, Finland.; 8 Department of Biomedical Engineering, School of Medicine, Emory University, Atlanta, Georgia.; 9 National Center for Cancer Immune Therapy, Department of Oncology, Copenhagen University Hospital, Herlev, Denmark.

## Abstract

The successful use of expanded tumor-infiltrating lymphocytes (TIL) in adoptive TIL therapies has been reported, but the effects of the TIL expansion, immunophenotype, function, and T cell receptor (TCR) repertoire of the infused products relative to the tumor microenvironment (TME) are not well understood. In this study, we analyzed the tumor samples (*n* = 58) from treatment-naïve patients with renal cell carcinoma (RCC), “pre-rapidly expanded” TILs (pre-REP TIL, *n* = 15) and “rapidly expanded” TILs (REP TIL, *n* = 25) according to a clinical-grade TIL production protocol, with single-cell RNA (scRNA)+TCRαβ-seq (TCRαβ sequencing), TCRβ-sequencing (TCRβ-seq), and flow cytometry. REP TILs encompassed a greater abundance of CD4^+^ than CD8^+^ T cells, with increased LAG-3 and low PD-1 expressions in both CD4^+^ and CD8^+^ T cell compartments compared with the pre-REP TIL and tumor T cells. The REP protocol preferentially expanded small clones of the CD4^+^ phenotype (*CD4*, *IL7R*, *KLRB1*) in the TME, indicating that the largest exhausted T cell clones in the tumor do not expand during the expansion protocol. In addition, by generating a catalog of RCC-associated TCR motifs from >1,000 scRNA+TCRαβ-seq and TCRβ-seq RCC, healthy and other cancer sample cohorts, we quantified the RCC-associated TCRs from the expansion protocol. Unlike the low-remaining amount of anti-viral TCRs throughout the expansion, the quantity of the RCC-associated TCRs was high in the tumors and pre-REP TILs but decreased in the REP TILs. Our results provide an in-depth understanding of the origin, phenotype, and TCR specificity of RCC TIL products, paving the way for a more rationalized production of TILs.

**Significance:**

TILs are a heterogenous group of immune cells that recognize and attack the tumor, thus are utilized in various clinical trials. In our study, we explored the TILs in patients with kidney cancer by expanding the TILs using a clinical-grade protocol, as well as observed their characteristics and ability to recognize the tumor using in-depth experimental and computational tools.

## Introduction

Renal cell carcinoma (RCC) is considered as a highly immunogenic cancer with an abundance of tumor-infiltrating lymphocytes (TIL; refs. 1–3). However, contrastingly to other cancers, the link between increased number of tumor CD8^+^ T cells and better patient prognosis is of debate in RCC (4–9). Approximately 30% of patients have metastatic disease at diagnosis, and the majority of the patients develop metastases later on (10, 11). Furthermore, immune checkpoint inhibitor (ICI) therapy leads to remission in only a proportion of patients with RCC (1, 12).

The reinfusion of TILs in the clinical setting has resulted in striking clinical responses in patients with metastatic melanoma with the combination of lymphodepletion and high-dose IL2 (13, 14). Adoptive TIL therapies have resulted in objective responses in almost half of the patients with metastatic melanoma (13, 15), including those refractory to anti-PD-1 therapy (16–18), encouraging the need for optimization in patients who fail other types of immunotherapies. Although long-lasting responses have been observed in patients with metastatic melanoma (17, 19), only one ongoing clinical trial using adoptive TIL therapy currently exists for RCC (NCT02926053; ref. 20), despite both tumors being known to respond to immunotherapies (21, 22). Past studies have reported weaker immune responses in RCC TILs than in metastatic melanoma (20, 23). However, a comprehensive understanding of the immunophenotype and function of the cells in the *ex vivo* expanded TIL product compared with the original tumor sample is still lacking.

In this study, we characterized the non-expanded and expanded TILs in patients with RCC and explored differences in the immune phenotype, tumor reactivity, cytotoxic ability, and T cell receptor (TCR) repertoires. With the use of the clinical-grade TIL expansion protocol that is currently applied in various clinical trials involving adoptive TIL therapies for patients with cancer (24–26), we compared the immunological differences between the minimally cultured “pre-rapid expansion” TILs (pre-REP TILs) and “rapidly expanded” TILs (REP TILs). We show our REP TIL protocol favors the expansion of CD4^+^ T cells, which originate from small T cell clones in the tumor microenvironment (TME). In contrast, large, expanded tumor CD8^+^ T cell clones mostly vanish during the TIL expansion. Furthermore, we identified RCC tumor-associated TCR motifs that were validated in multiple TCRβ-sequencing (TCRβ-seq) and single-cell RNA (scRNA)+TCRαβ-seq (TCRαβ sequencing) datasets. As a result, T cells carrying the RCC-associated TCR motifs were enriched in the pre-REP TIL samples, whereas the frequency was reduced in the REP TILs, suggesting that some tumor-reactive T cell clones are lost during the expansion.

## Material and Methods

### Patient Cohort and Study Approval

**FIGURE 1:**
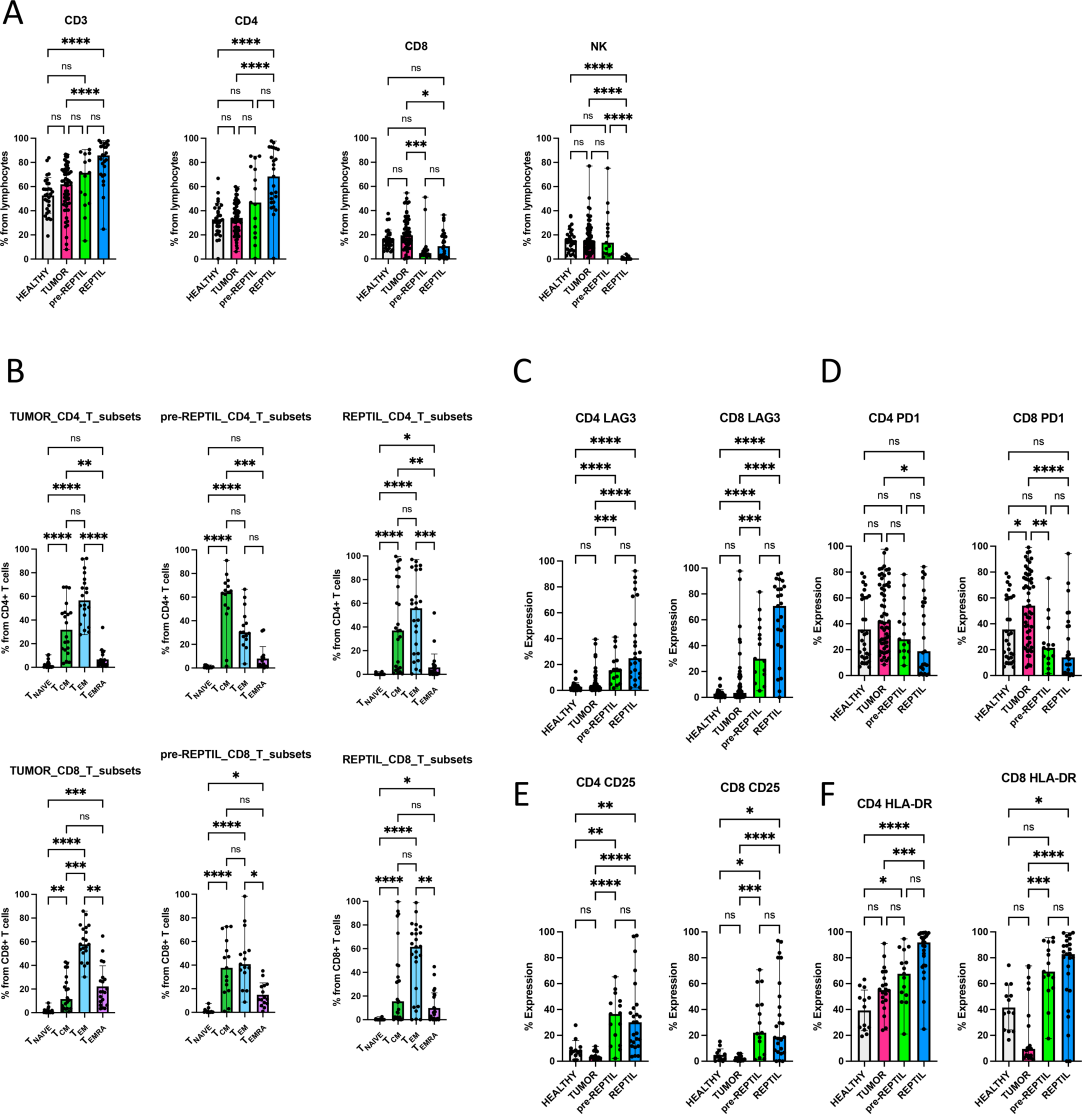
Immunophenotypic differences between tumor, healthy kidney tissue, pre-REP TILs, and REP TILs. **A,** Matching healthy kidney tissue (*n* = 30), tumor (*n* = 58), pre-REP TIL (*n* = 15), and REP TIL (*n* = 25) samples were used for flow cytometry immunophenotypic analysis. The median positive expression levels were compared between each sample type. The pre-REP TILs and REP TILs had a greater abundance of CD4^+^ T cells (median 46.8% and 68.4% out of lymphocytes, respectively) than CD8^+^ T cells (4.8% and 10.5%), compared with the tumor CD4^+^ (34%) and CD8^+^ (19.5%) T cells. In contrast, an increased proportion of cells in the tumor (14.4%), healthy kidney (14.4%), and pre-REP TIL (13.5%) samples were NK cells. **B,** The CD4^+^ and CD8^+^ T cell immunophenotypes were analyzed using CCR7 and CD45RO markers. Most of the pre-REP TIL CD4^+^ T cells were of the central memory (T_CM_; CCR7^+^ CD45RO^+^) phenotype (median 64%), and the pre-REP TIL CD8^+^ T cells were either T_CM_ (37.7%) or effector memory (T_EM_; CCR7-CD45RO^+^, median 40.8%) cells. REP TIL CD4^+^ T cells were T_CM_ (37%), but more T_EM_ (55.8%) cells, whereas more than half (61.5%) of the REP TIL CD8^+^ T cells were T_EM_ cells. T_NAIVE_ = naïve T cells, T_CM_ = central memory T cells, T_EM_ = effector memory T cells, T_EMRA_ = terminally differentiated effector memory T cells. **C,** The greatest LAG-3 expression was observed in the REP TIL CD8^+^ T cells (median 70.7%) compared with healthy kidney (1.6%), tumor (3.5%), and pre-REP TIL (30%) samples. **D,** PD-1 expression was greatest in the tumor CD4^+^ (41.6%) and CD8^+^ (54%) T cells, suggesting an exhausted phenotype compared with the healthy tissue, pre-REP TILs and REP TILs. **E,** CD25 expression was highest in both pre-REP TIL (36.5%) and REP TIL (30.1%) CD4^+^ T cells compared with the tumor and healthy kidney. **F,** The highest HLA-DR expression was observed in the pre-REP TILs and REP TILs in both CD4^+^ (67.6% and 91.9%, respectively) and CD8^+^ T cell compartments (69.3% and 82.8%). For all immunophenotypic analyses, Kruskal–Wallis nonparametric test with Dunn’s *post hoc* test was used for all analyses. ns, not significant; *, *P* < 0.05; **, *P* < 0.01; ***, *P* < 0.001; ****, *P* < 0.0001.

The study included 58 patients with RCC undergoing radical nephrectomy between 2016 and 2020 (Fig. 1A). Primary tumor tissue (*n* = 58), adjacent healthy kidney tissue (*n* = 30), and matched peripheral blood (PB; *n* = 40) samples were obtained. All procedures were conducted in compliance with the Declaration of Helsinki and the studies were approved by the Helsinki University Hospital Ethical Committee (Dnro 115/13/03/02/15) and. All samples were obtained after a written informed consent.

### Clinical Data

In total, 18 clinical parameters including tumor size, World Health Organization International Society of Urologic Pathologists (ISUP) 2016 grade, tumor–node–metastasis (TNM) staging, presence of necrosis, perirenal and peripelvic fat infiltration, rhabdoid histology, and other medical histories were assessed (Supplementary Table S1).

### Sample Processing

Briefly, the tumor and healthy kidney tissue samples were stored in MACS tissue storage solution (Miltenyi Biotec 130-100-008) at 4°C upon harvest and processed immediately upon arrival using Miltenyi's Tumor Dissociation kit (Miltenyi Biotec 130-095-929). The remaining dissociated cells were viably frozen in 10% FBS-DMSO freezing solution and kept at −150°C until further use. Peripheral blood mononuclear cells (PBMC) were separated from PB samples with density gradient centrifugation using Ficoll-Paque (GE Healthcare) and viably frozen in 10% FBS-DMSO at −150°C.

### Generation of pre-REP and REP TIL Cultures

pre-REP TILs were isolated *in vitro* from whole tumor fragments (1–2 mm^3^) as described previously (27). Briefly, each tumor fragment was placed into a single 24-well plate supplemented with TIL media: 90% RPMI1640 (Corning 15303561), 10% heat-inactivated human AB serum (Merck H3667), 6,000 IU/mL IL2 (Bio-Techne 202-IL-500), 1% penicillin-streptomycin (Gibco, 15140122), and 1.25 μg/mL Amphotericin B (Gibco 15290018). The fragments were cultured for 3 weeks to collect the *in vitro* expanded pre-REP TILs. The pre-REP TILs were pooled and counted using Trypan Blue and Bürker chamber. Next, a proportion of the pre-REP TILs (minimum 100,000 cells) was expanded using a 14-day “rapid expansion” (REP) protocol, as previously outlined by Andersen and colleagues (20). Briefly, 100,000 pre-REP TILs were cultured in the presence of 20 million feeder cells consisting of irradiated PBMCs pooled from 8 different healthy donors (Finnish Red Cross), the anti-CD3 (OKT3) antibody (30 ng/mL, Miltenyi Biotec 170-076-116), and IL2 (6,000 IU/mL, Bio-Techne 202-IL-500) for the expansion of T cells. PBMCs for the feeder cells were isolated by density gradient centrifugation using Ficoll-Paque (GE Healthcare). The complete pre-REP TIL and REP TIL protocols have been previously described in detail (27).

### Multi-parameter Flow Cytometry and Immunophenotyping

Freshly dissociated tumor, healthy kidney, and TIL samples were used to examine the immune cell numbers and immunophenotypes. The samples were stained for 15 minutes with a comprehensive antibody staining panel as described previously (28) and were washed twice with PBS before phenotyping. A total of 50,000 lymphocytes were acquired per tube with FACS Verse (BD Biosciences) and analyzed with FlowJo (Version 10.0.8rl, Treestar). A full list of markers is provided in Supplementary Table S2. All antibodies were purchased from BD Biosciences unless mentioned otherwise. In addition, the availability of samples used in each analysis is provided in Supplementary Table S3.

### REP TIL and Tumor Co-cultures

Autologous *in vitro* cultured two-dimensional tumor cells (derived from the same lesions as the TIL cultures) were established from live frozen tumor dissociated cells using the Primary Cancer Culture System kit (PromoCell C-28081) in 12-well plates. The morphology and growth patterns of the tumor cells were examined using light microscopy. *Mycoplasma* tests (MycoAlert PLUS, Lonza LT07-710) were performed and were negative. After 4–7 weeks of cell culture maintenance, cells were checked for 90%–100% confluency and used for the co-cultures. The cocultures included a 6-hour and 48-hour incubation period, whereby 1 million REP TILs were incubated with the corresponding tumor cells for both timepoints. GolgiStop was added 6 hours before the end of the experiment to measure intracellular cytokine production. The TILs were gently pipetted from the wells and phenotyped using the FACS Verse (BD Biosciences). The data were analyzed with FlowJo (v.10.8.1, Treestar). All antibodies were purchased from BD Biosciences unless mentioned otherwise. The full list of the antibodies and catalog numbers is provided in Supplementary Table S2, together with details concerning the usage of the samples in Supplementary Table S3.

### REP TIL T cell Activation Assay

REP TILs were resuspended in RPMI (10% FBS, penicillin, 1% streptomycin, and 1% l-glutamine; Lonza) and stimulated with anti-CD3 (clone UCHT1), anti-CD28, anti-CD49d, in the presence of GolgiStop, CD107a and CD107b antibodies (CD107a/b). After 6 or 48 hours of incubation at 37°C, the cells were harvested and washed. Next, the cells were stained with the following antibodies: CD45, CD3 (clone SK7), CD4, CD8, and CD56. After staining the surface markers, cells were fixed and permeabilized with Fix/Perm (BD Biosciences, 554714) according to the manufacturer's instructions. The intracellular markers TNFα, IFNγ, and Granzyme B (GzB) were next stained for. A total of 50,000 CD45^+^ cells were acquired/tube using FACS Verse (BD Biosciences) and analyzed with FlowJo (v.10.8.1, Treestar). The full list of antibodies and catalog numbers is provided in Supplementary Table S2, together with details of the sample usage in Supplementary Table S3.

### Bulk TCRβ-seq Data Acquisition and Analysis

Bulk TCRβ-seq was carried out from genomic DNA using the Adaptive Biotechnologies ImmunoSEQ Assay “Survey” resolution (29). Only productive (complete, in-frame) TCRβ rearrangements were included in the analysis. Downstream analyses were performed with VDJTools (ref. 30; v.1.2.1) and immunarch (ref. 31; v.0.6.7); non-functional clonotypes were removed and samples were downsampled to the smallest repertoire in each sample type. Samples with less than 10,000 reads were excluded from further analysis. Multiple diversity metrics (Inverse Simpson, Chao1, and Gini) and clonality indices were calculated for both the total and downsampled datasets.

Expanded clonotypes between the pre-REP TIL and REP TIL samples were defined from all datasets (no downsampling) using Fisher exact test (two-sided), with Benjamini-Hochberg–corrected *P* values. Adjusted *P* values <0.05 were considered significant (expanded clonotype). Epitope-specific TCR predictions were performed using TCRGP (ref. 32; v.1.0.0), with anti-viral and CDR3β models.

### scRNA+TCRαβ-seq Data Acquisition and Analysis

Viable frozen tumor dissociated cells from 3 patients with RCC were thawed in PBS, 2 mmol/L Ethylenediaminetetraacetic acid (EDTA), and the CD45^+^ cells were selected with the Sony SH800 cell sorter (Sony Biotechnology Inc.) for sequencing. Next, single cells were partitioned into the Chromium Controller (10X Genomics). The scRNA-seq+TCRαβ libraries were prepared using the Chromium Single Cell 5′ Library & Gel Bead Kit (v. 1.1, 10X Genomics) according to the manufacturer's instructions (CG000086 Rev D) and as described previously (33, 34).

Briefly, 12,000 cells from each sample were resuspended in 0.04% BSA-PBS solution and loaded onto the Chromium Single Cell A Chip. Single-cell barcoded cDNA were produced, and the remainder of the steps were performed according to the manufacturer's instructions. A total of 14 PCR cycles in the Vetri thermal cycler (Applied Biosystems) were run to amplify the full-length cDNA. Subsequently, the Chromium Single Cell Human T Cell V(D)J Enrichment Kit (10X Genomics) was used to amplify the TCR cDNA. The Illumina NovaSeq 6000 S1 Flow Cell [read length configuration: 26 bp (Read 1), 8 bp (i7 Index), 0 bp (i5 Index), and 91 bp (Read 2)] was used to sequence the gene expression libraries with a sequencing depth of 50,000 read pairs/cell; the Illumina HiSeq2500, Rapid Run mode (read length configuration: Read1  =  150, i7  =  8, i5  =  0, Read2  =  150) was used to sequence the TCR-enriched libraries with a sequencing depth of 5,000 read pairs/cell. The raw data were preprocessed with the Cell Ranger (v.3.1) software and aligned with the human GRCh38 reference genome. The scRNA-seq+TCRαβ-seq data were combined and analyzed to assess the phenotype of the expanded pre-REP TIL and REP TIL clonotypes from the tumor (*n* = 3) using scvi-tools (ref. 35; v.0.13.0), Seurat (refs. 36, 37; v.4.1.0), and scRepertoire (38).

### scRNA+TCRαβ-seq Data Analysis

The scRNA-seq+TCRαβ-seq data were analyzed to assess the phenotype of the expanded pre-REP TIL and REP TIL clonotypes from the tumor (*n* = 3). Quality control metrics were assessed individually for each sample by visual inspection and low-quality cells were additionally removed in further stages of the analysis when identified. We combined the samples and utilized scvi-tools (ref. 35; v.0.13.0) for batch correction using default parameters, treating each sample as one batch and correcting for cell-cycle heterogeneity by using scores calculated with the Seurat “CellCycleScoring” function as covariates. The latent embeddings were used for graph-based clustering and uniform manifold approximation and projection (UMAP) dimensionality reduction in Seurat (refs. 36, 37; v.4.1.0) with default parameters. In addition, we used scRepertoire (38) to combine the TCRαβ-seq data with the Seurat object, where individual clonotypes were defined with the “CTstrict” criteria, meaning that a clonotype should share exactly the same nucleotide sequence of the TCR.

For clustering, we visually inspected the results by varying the “resolution” parameter in the “FindClusters” function between 0.2 and 0.5. This allowed us to find the optimal number of clusters without overclustering or underclustering, based on the agreement between UMAPs and the chosen clustering resolution, together with whether we could find biological phenotypes for each cluster. Clusters were named and annotated by analysis of canonical markers (39, 40), differentially expressed genes (DEGs), relationship to other clusters, and TCR repertoire clonalities. The default parameters in the “RunUMAP” function were used for all UMAP dimensionality reductions. The “AddModuleScore” functions, as defined by Tirosh and colleagues (41), were used to calculate the different scores based on the expression of specific genes. The cytotoxicity score was calculated using the genes *GZMB, GZMA, GZMH, PRF1, GNLY,* and *FGFBP2*. The exhaustion score was calculated on the basis of *PDCD1, LAG3, CTLA4, TIGIT, TOX,* and *HAVCR2*. We used *ITGA1, ITGAE, ZNF683, CD69, IFNG,* and *CCR5* to calculate the tissue resident score. scRepertoire (38) was used to combine the TCRαβ-seq data with the Seurat objects. DEG analyses were performed with Wilcoxon rank-sum test and *P* values were Bonferroni adjusted and corrected. Genes with adjusted *P* values <0.05 were considered significant. Fisher exact test with a two-sided alternative was used to study the overlapping TCRs between the pre-REP TILs and REP TILs.

Epitope-specific TCR predictions were performed using TCRGP (ref. 32; v.1.0.0), with anti-viral models (Influenza A M1_GILGFVFTL_, EBV BMLF1_GLCTLVAML_, CMV pp65_IPSINVHHY_, CMV pp65_NLVPMVATV_, EBV BZLF1_RAKFKQLL_, CMV pp65_TPRVTGGGAM_, and EBV BRLF1_YVLDHLIVV_ epitope, and SARS-CoV2 S1_YLQPRTFLL_) and CDR3β models gathered from the TCRGP GitHub page (https://github.com/emmijokinen/TCRGP). For the predictions used in all analyses, a false-positive threshold rate of 5% was determined for each epitope separately from the ROC curves obtained from the cross-validation experiments in the original publication (32).

### Identification and Validation of RCC-associated Motifs

The GLIPH2 (42) algorithm (v.1.0.0) was run separately on the discovery cohorts of the pooled tumor (*n* = 42), healthy kidney (*n* = 24), and PB (*n* = 32) TCRβ-seq samples using default parameters. The RCC-associated motifs consisted of those that were exclusive and enriched to the tumor. Tumor-exclusive motifs were found only in the tumor samples after filtering. Motifs that were shared between the tumor-healthy kidney and tumor-PB were analyzed using the two-sided Wilcoxon test; all TCRs with a motif were pooled together, and TCRs without motifs were noted as 0. *P* values were corrected with Benjamini-Hochberg adjustment and only motifs with an adjusted *P* value <0.05 and log_2_fc >1 were annotated as tumor-enriched. The RCC-associated motifs are listed in Supplementary Table S4.

### Validation of RCC-associated Motifs

GLIPH2 (ref. 42; v.1.1.0) was run on validation cohorts that included CD4^+^ and CD8^+^ sorted T cell fractions obtained from healthy donor PB samples (refs. 33, 43; *n* = 37), CD8^+^ sorted fractions (ref. 44; *n* = 37) from patients with rheumatoid arthritis, and epitope-specific data from VDJdb (45) consisting of 80 different viral motifs. Clusters with at least three different TCRs and motifs with a minimum length of three were retained. Motifs found in the validation cohort were removed from the discovery cohort to increase the signal-to-noise ratio.

### Analysis of RCC-associated Motifs in Different TCRβ-seq and scRNA+TCRαβ-seq Datasets

#### Bulk TCRβ-seq

RCC-associated motifs were analyzed in different cohorts, including the discovery cohort (42 tumor, 24 healthy kidney, and 32 PB samples), validation cohorts (seven pre-REP TIL samples and seven matching REP TIL samples), and comparison cohorts [Emerson and colleagues (46): 786 PB samples from healthy donors; Huuhtanen and colleagues (47): 226 tumor samples from patients with metastatic melanoma; The Cancer Genome Atlas (TCGA; ref. 48): 1,190 tumor samples from various tumors]. Motifs had to be found in the same location of the CDR3 regions as in the original GLIPH2 run. From PB samples (RCC and healthy donor cohorts), only the non-singleton TCRs were included (singleton referring to a TCR with one read). From TCGA cohort (48), only samples with at least 100 TCR reads were included. From the healthy donor cohort (46), samples were downsampled to 40,000 reads/sample to allow reliable comparisons with the RCC samples.

#### scRNA+TCRαβ-seq

RCC-associated motifs were examined in different cohorts, including the discovery cohort (three tumor samples) and validation cohorts [Zheng and colleagues (49): 82 samples from various tumors; Krishna and colleagues (50): 29 samples from 6 patients with RCC multi-region sampling from tumor, healthy kidney, PB, and lymph nodes]. Motifs had to be found in the same location in the TCRβ chain as in the original GLIPH2 run. From the Zheng and colleagues (49) cohort, only samples with at least 100 TCR reads were included.

### Immunophenotype of RCC-associated Motifs in scRNA+TCRαβ-seq Data

In the validation cohorts, the phenotypes were obtained from the original publications (49, 50). For each pattern with at least five supporting cells, the odds ratio (OR) for a pattern with a certain phenotype was calculated, and the phenotype with the largest OR was assumed to be the dominant phenotype.

### Statistical Analysis

Non-parametric Wilcoxon matched-pairs signed rank test was used for paired analyses. Non-parametric Kruskal–Wallis test with Dunn’s *post hoc* test and a familywise alpha threshold, confidence level 0.05 was used to compare different sample types (healthy, tumor, pre-REP TIL, REP TIL) with GraphPad Prism (v.9.4.0). Two-sided Fisher’s exact test with Benjamini-Hochberg adjusted *P* values <0.05 was used. For boxplots, the center line corresponds to the median, the box corresponds to the interquartile rage (IQR), the whiskers to 1.5xIQR, and outlier points were individually plotted where present. All other computational analyses were performed using R (v.4.0.2) (R Core Team (2021). R: A language and environment for statistical computing. R Foundation for Statistical Computing, Vienna, Austria. Available from: https://www.R-project.org/), RStudio (v.2022.02.3) (RStudio: Integrated Development for R. RStudio, PBC. Available from: http://www.rstudio.com/), and Python (v.3.7.4). For all graphs: ns, not significant; *, *P* < 0.05; **, *P* < 0.01; ***, *P* < 0.001; ****, *P* < 0.0001.

### Data Availability

The raw bulk TCRβ-seq data are available from immuneAccess (DOI: 10.21417/MHL2023CRC). The scRNA+TCRαβ-seq data are available as processed in BioStudies, ArrayExpress (E-MTAB-12910) and as raw data in the European Genome-Phenome Archive (EGAS00001006952S). The Seurat objects are available in Zenodo (10.5281/zenodo.7386294).

## Results

### RCC Tumors, Healthy Tissue, Pre-REP TILs, and REP TILs Display Immunophenotypic Differences

In total, we collected 58 primary tumor samples (Supplementary Fig. S1; Supplementary Table S3). From 25 unselected cases from which we had enough sample material available, we aimed to expand the pre-REP TILs and REP TILs. The expansion protocol was successful in all cases, resulting in a total of 25 REP TIL samples. However, in 10 patients, the number of pre-REP TILs was limited, and we could only use these cells for the REP protocol. Thus, for the downstream immunophenotype analyses, we utilized 15 pre-REP TIL and 25 REP TIL samples (Supplementary Fig. S1; Supplementary Table S3 includes the number of pre-REP and REP TILs received in each case). In addition, from 58 tumor cases, 30 matching healthy tissue samples were available and thus used for the flow cytometry analysis (Supplementary Table S3). Representative flow cytometry gating strategies for the various sample types, marker expressions, and paired co-culture assays described below are presented in Supplementary Fig. S2–S5. The initial median proportion of lymphocytes from all dissociated tumor cells was 1.6% compared with 0.69% in the healthy kidney samples. During the clinical grade TIL expansion protocol, T cells were massively expanded; over 80% of the lymphocytes were CD3^+^ T cells in the REP TILs and only few natural killer (NK) cells (0.03%) were observed (Fig. 1A). The overall median proportion of lymphocytes was 89.2% in the pre-REP TILs and 94.9% in the REP TILs (Supplementary Fig. S6A). In contrast, an increased proportion of cells in the tumor (14.4%), healthy kidney (14.4%), and pre-REP TIL (13.5%) samples were NK cells (Fig. 1A). In the tumor samples, CD4^+^ T cells were already more prevalent than CD8^+^ T cells (34% vs. 19.5%), and in pre-REP TILs and REP TILs, the CD4^+^ T cell dominance further increased (46.8% and 68.4%, respectively; Fig. 1A).

We next analyzed the T cell immunophenotypes between the different samples using the expression of CCR7 and CD45RO markers. Both tumor and healthy CD4^+^ and CD8^+^ T cells predominantly displayed an effector memory (T_EM_) phenotype (Fig. 1B; Supplementary Fig. S3B). Among the pre-REP TILs, CD4^+^ T cells mostly displayed a central memory (T_CM_) phenotype (64%), whereas CD8^+^ T cells had both T_CM_ (37.7%) and T_EM_ (40.8%) cells in similar quantities. In the REP TILs, 37% of CD4^+^ T cells had a T_CM_ phenotype and approximately half were T_EM_ cells (55.8%), whereas more than half the CD8^+^ T cells were predominantly T_EM_ cells (61.5%; Fig. 1B).

Next, we analyzed clinically relevant immune checkpoint markers (LAG-3 and PD-1) and found that LAG-3 was significantly increased in both REP TIL CD4^+^ (median 24.9%) and CD8^+^ T cells (70.7%) than in the pre-REP TILs (15.5% and 29.9%) and tumors (2.7% and 3.5%; Fig. 1C). In contrast, PD-1 expression decreased in both REP TIL CD4^+^ (18.8%) and CD8^+^ T cells (14%) compared with the pre-REP TILs (28% and 21.4%) and tumors (41.6% and 54%), indicating that T cells residing in the tumor encompassed the greatest amount of exhaustion (Fig. 1D). Accordingly, in both the pre-REP TILs and REP TILs, CD25 and HLA-DR expressions were increased in the CD4^+^ and CD8^+^ T cells, suggesting the activation of T cells during the expansion protocol (Fig. 1E and F).

From the limited number of cases, further analyses with paired sample types were available (Supplementary Fig. S6C and S6D). Because of the small sample size, differences between the groups were not significant, but similar trends (increased LAG-3 and HLA-DR, decreased PD-1 expression) were observed (Supplementary Fig. S6C and S6D).

### The Expansion Potential of TILs Correlates with CD4^+^ T_CM_ Phenotype

To understand whether the expansion potential was correlated with a distinct cell type in the product, we calculated the fold change between the number of REP TILs at the end of the expansion protocol and the starting number of pre-REP TILs (Supplementary Table S3), then compared the fold change with the immunophenotyping data. A total of nine pre-REP TIL samples with available information were compared for their expansion capacities (Supplementary Fig. S6E–S6G; Supplementary Table S3). A negative correlation was observed between the pre-REP TIL CD4^+^ T_CM_ cells and the REP TIL/pre-REP TIL fold-change (Spearman, *R* = −0.73, *P* = 0.03; Supplementary Fig. S6E), suggesting that a low amount of CD4^+^ T_CM_ cells initially in the pre-REP TILs led to a better yield of REP TILs. Accordingly, a higher quantity of CD4^+^ T_EM_ cells in the pre-REP TILs led to a better REP TIL expansion although no statistical significance was observed (Spearman, *R* = 0.67, *P* = 0.059). A similar negative trend was observed between the pre-REP TIL CD8^+^ T_CM_ cell phenotype and REP TIL/pre-REP TIL fold-change (Supplementary Fig. S6F). In addition, a borderline negative correlation between the pre-REP TIL CD4^+^ T cells and PD-1 expression and REP TIL/pre-REP TIL fold-change was observed (Spearman, *R* = −0.62, *P* = 0.06), suggesting that putatively exhausted CD4^+^ T cells expressing the marker do not expand well in the REP protocol (Supplementary Fig. S6G).

### REP TIL CD4^+^ and CD8^+^ T cells Respond to Tumor Cells in Coculture Models

**FIGURE 2:**
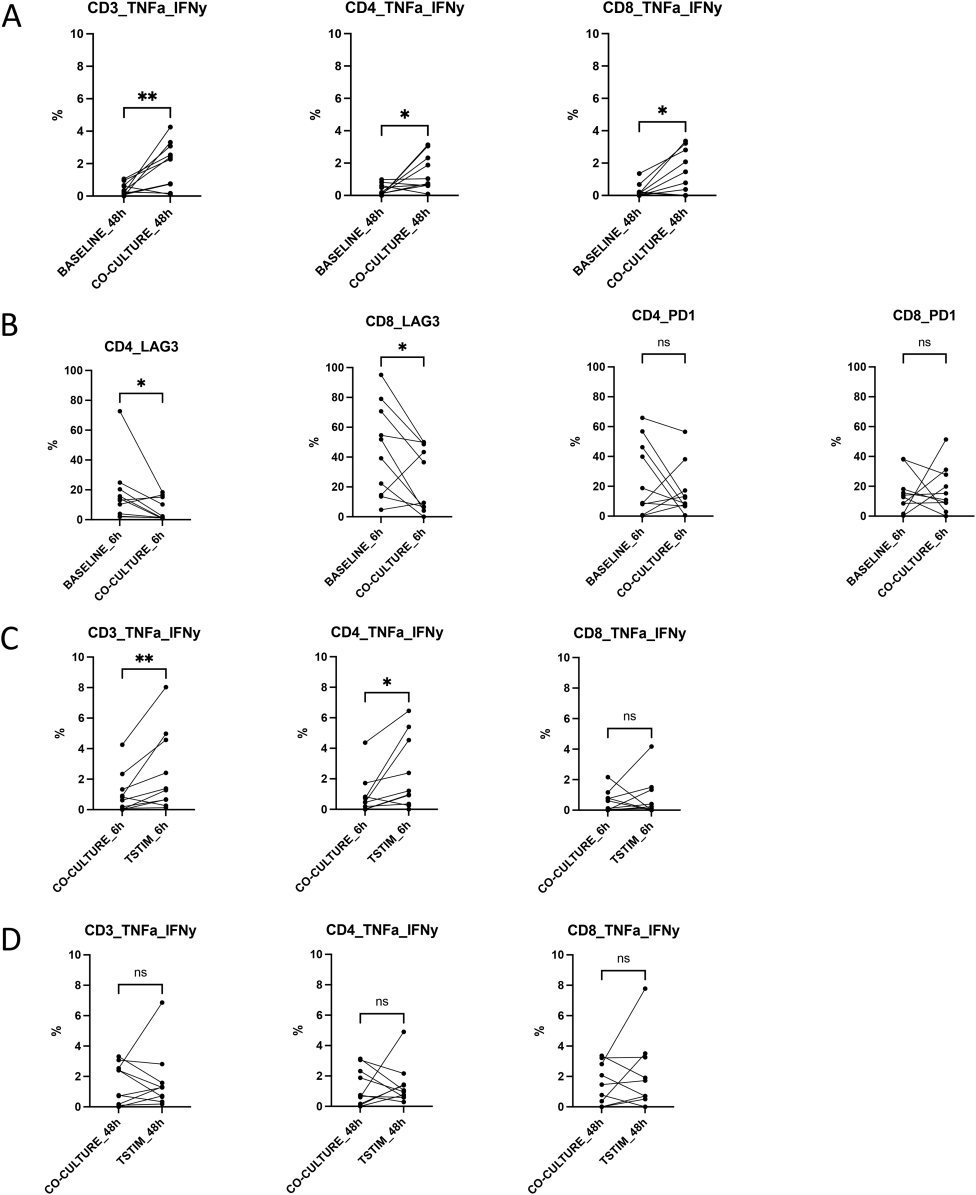
REP TIL T cells co-cultured with tumor cells show differences in IFNγ and TNFα expressions. **A,** REP TILs were cocultured with the corresponding tumor cells (*n* = 10) either for 6 or 48 hours. The cytokine secretion of CD4^+^ and CD8^+^ T cells was analyzed with intracellular flow cytometry staining. The expression of IFNγ and TNFα moderately increased in REP TILs after the co-culture with tumor cells without additional T cell stimulation. After 48 hours co-culture, IFNγ and TNFα levels were increased in both CD4^+^ (0.74% vs. 0.19%, *P* = 0.044) and CD8^+^ T cells (0.78% vs. 0.13%, *P* = 0.025) compared with baseline REP TILs without tumor cell co-culture. No differences were observed at 6 hours co-cultures (Supplementary Fig. S3A). BASELINE_48h = only REP TILs at 48h, CO-CULTURE_48h = cocultured REP TILs without any stimulation at 48 hours. **B,** The immunophenotype of T cells after co-culture with tumor cells was analyzed with flow cytometry. The expression of LAG-3 moderately decreased in the CD4^+^ (median 13.55% vs. 1.84%, *P* = 0.048) and CD8^+^ T cells (45.55% vs. 22.94%, *P* = 0.029) compared with REP TIL baseline cells at 6 hours. The same trends were not observed when REP TILs were co-cultured for 48 hours (Supplementary Fig. S3D). Although not significant, the expression of PD-1 expression increased in half of the patients. BASELINE_6h = only REP TILs at 6h, CO-CULTURE_6h co-cultured REP TILs without any stimulation at 6 hours. **C,** T cell activation potential was assessed by stimulating the cells with anti-CD3 (OKT3), anti-CD28, anti-CD49d antibodies, and IFNγ and TNFα cytokine secretion was measured as described above. When REP TILs were stimulated and co-cultured for 6 hours, an increase in the CD3^+^ T cell IFNγ and TNFα expressions was observed between unstimulated and stimulated co-cultures (median 0.72% vs. 1.33%, *P* = 0.098). A moderate increase in CD4^+^ T cell IFNγ and TNFα expressions was also observed (0.46% 1.089%, *P* = 0.012), but not in the CD8^+^ T cells. CO-CULTURE_6h = cocultured REP TILs without any stimulation at 6h, TSTIM_6h = cocultured REP TILs that were T cell stimulated at 6 hours. **D,** Prolonged co-culture conditions (48 hours) did not lead to increased IFNγ and TNFα production in the CD3^+^, CD4^+^, and CD8^+^ T cell compartments. CO-CULTURE_48h = cocultured REP TILs without any stimulation at 48h, TSTIM_48h = cocultured REP TILs that were T cell stimulated at 48 hours. Non-parametric Wilcoxon matched-pairs signed rank test was used for all co-culture analyses. ns, not significant; *, *P* < 0.05; **, *P* < 0.01; ***, *P* < 0.001; ****, *P* < 0.0001.

To explore how REP TILs react when exposed to tumor cells, we set up a coculture system by incubating (6 and 48 hours) the REP TILs with the primary tumor cells of the same patient (*n* = 10). First, we measured the ability of the REP TILs to produce the cytokines IFNγ and TNFα when cocultured with the tumor cells. Compared with REP TIL baseline measurements, we observed a moderate increase in IFNγ and TNFα expression in the CD3^+^ (median 0.34% vs. 2.27%), CD4^+^ (0.19% vs. 0.74%), and CD8^+^ (0.13% vs. 0.78%) T cells after 48 hours of co-culture (Fig. 2A), but not at 6 hours (Supplementary Fig. S7A), suggesting that REP TILs increase their tumor reactivity with prolonged exposure to the matching tumor cells. However, no differences were observed in CD107a/b and GzB expressions after 6 and 48 hours cocultures (Supplementary Fig. S7B and S7C). LAG-3 expression decreased in CD4^+^ (13.55% vs. 1.84%, *P* = 0.048) and CD8^+^ T cells (45.55% vs. 22.94%, *P* = 0.029) after 6 hours of co-culture with tumor cells (Fig. 2B), but similar trends were not observed at 48 hours of co-culture (Supplementary Fig. S7D). The changes in PD-1 expression upon co-culture varied between individuals, and in half of the patients, PD-1 expression increased (Fig. 2B).

We also stimulated T cells with anti-CD3 and the costimulatory antibodies, anti-CD28 and anti-CD49d to activate T cell cytokine production and degranulation. REP TILs were able to respond to activation in the presence of tumor cells; increases in the CD3^+^ (median 0.72% vs. 1.33%, *P* = 0.098) and CD4^+^ (0.46% vs. 1.08%, *P* = 0.012) T cell IFNγ and TNFα expressions were observed after 6 hours of co-culture (Fig. 2C). However, after prolonged co-culture conditions (48 hours), anti-CD3^+^, anti-CD28^+^, and anti-CD49d^+^ stimulation of the T cells no longer led to increased cytokine production, suggesting either that the immunosuppressive role of the tumor cells, or cytokine production had already reached its maximum, with only tumor cells as a stimulus to the T cells (Fig. 2D). Furthermore, a moderate decrease in CD8^+^ T cell GzB expression (*P* = 0.039) at 6 hours was observed, with a borderline increase in the expression of CD107a/b in CD8^+^ T cells at 6 and 48 hours of T cell stimulation (*P* = 0.063; Supplementary Fig. S7E and S7F).

### Bulk TCRβ-seq Shows Differences Between Tumor and TIL T cell Repertoires

**FIGURE 3:**
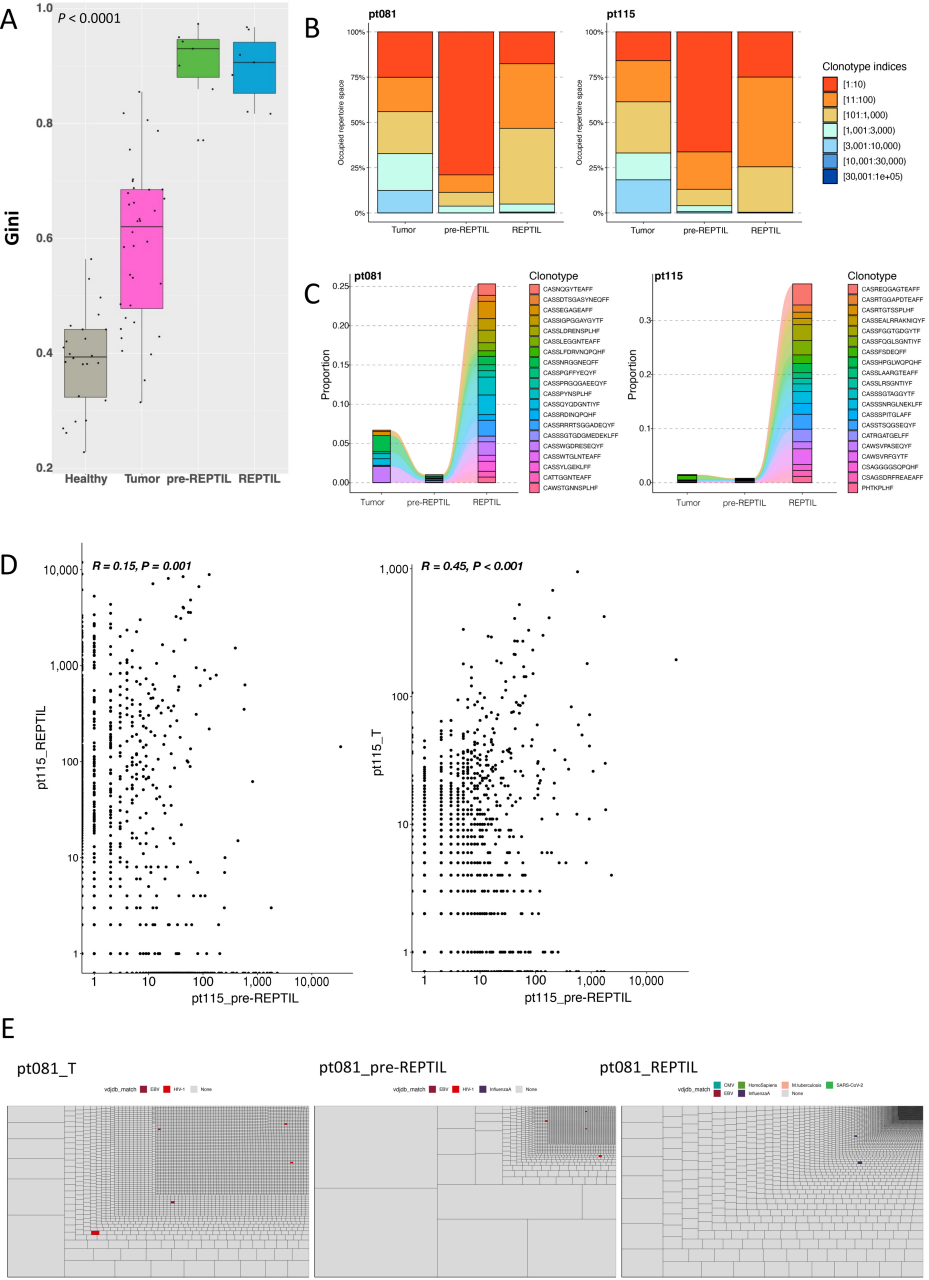
T cell repertoire differences between the tumor and TIL T cells. **A,** Healthy kidney (*n* = 24), tumor (*n* = 36), pre-REP TIL (*n* = 7), and REP TIL (*n* = 7) samples were sequenced with bulk TCRβ-seq. The clonality of the TCR repertoire was analyzed with the Gini index (values closer to 1 denote increased clonality). The pre-REP TILs were observed to be the most clonal out of the different samples, suggesting the loss of TCR repertoire diversity during the REP protocol. *P* < 0.0001, Kruskal–Wallis nonparametric test. **B,** Two representative cases (pt081 and pt115) showing the relative abundance of clonotypes in the tumor, pre-REP TILs, and REP TILs. The pre-REP TILs were dominated by the top 10 clones of the entire repertoire. Clonotype indices represent the rank of the clone (i.e., 1:10 indicates the top 10 most expanded clonotypes). Comparisons between the rest of the samples are found in Supplementary Fig. S4A. **C,** The top 20 most abundant REP TIL clonotypes were tracked to the corresponding tumor and pre-REP TIL samples in the same representative cases (pt081 and pt115). Overall, the clonotypes ending up in the REP TILs were very small clonotypes in the other sample types. Comparisons between the rest of the samples are found in Supplementary Fig. S4B. **D,** The frequencies of the overlapping T cell clonotypes in one representative case (pt115) between the REP TIL and pre-REP TIL samples (left), as well as between the tumor and pre-REP TILs (right). Spearman correlation was used to compare the frequencies of the clonotypes in the samples. The *x*- and *y*-axes are logarithmically transformed. *R* and *P* values refer to the log-transformed frequencies of the clonotypes. The frequencies for the rest of the samples are found in Supplementary Fig. S5 and S6). **E,** Treemaps showing the size of the T cell clonotypes in each sample type (tumor, pre-REP TIL, and REP TIL) in one representative case (pt081). The size of each box indicates the size of one clonotype in the TCR repertoire. Colored boxes indicate TCRs matched to viral-specific TCRs found in VDJdb (45). Few matches were discovered and represented as small T cell clonotypes, indicating that expanded clones in the tumor, pre-REP TILs and REP TILs do not target common viruses. Treemaps for all the samples are found in Supplementary Fig. S7.

First, we analyzed the clonality between the TCR repertoires of the healthy kidney (*n* = 24), tumor (*n* = 36), pre-REP TIL (*n* = 7), and REP TIL (*n* = 7) samples using the Gini index, a measure of clonal inequality (values closer to 1 denote higher clonality). The pre-REP TILs and REP TILs were significantly more clonal than the tumor and healthy kidney samples, suggesting that the TCR repertoire diversity was lost during the expansion protocol (Fig. 3A). When we further explored the differences between the TCR repertoires of the various samples, we observed that the pre-REP TIL TCR repertoire was mostly dominated by the top 10 most abundant clones, whereas in the tumors, the repertoire was more variable, comprised of smaller clones (Fig. 3B; Supplementary Fig. S8AI–S8V). To better understand what kinds of tumor T cell clonotypes were expanded in the REP TILs, we tracked the top 20 most abundant REP TIL clonotypes (CDR3 amino acid sequences) in the matching tumor, pre-REP TILs and REP TILs from seven samples (three samples additionally had healthy kidney data). In most cases, the top 20 REP TIL clonotypes comprised approximately 5% of the entire TCR repertoire in the tumor sample (Fig. 3C; Supplementary Fig. S8BI–S8V). Overall, the clonotypes enriched in the REP TILs were found to be very small clones in the matching samples. Our results were confirmed when we analyzed the total clonal overlap between REP TILs and pre-REP TILs, as well as between the tumor and pre-REP TILs in terms of the total number of clones in each sample repertoire (Fig. 3D; Supplementary Fig. S9 and S10). To understand whether the expanded clonotypes target common viral antigens, we matched the TCRs to VDJdb (45), a publicly curated TCR database. Individual matches to numerous viral epitopes (such as CMV, EBV, Influenza-A, and HIV-1) were discovered; however, these clonotypes were few and comprised only a small proportion of the entire TCR repertoire, suggesting that the expanded clones either in the tumor, pre-REP TILs, and REP TILs did not target common viruses (Fig. 3E; Supplementary Fig. S11).

### Identification and Validation of RCC-associated TCR Motifs in Large TCRβ-seq and scRNA+TCRαβ-seq Sample Cohorts

**FIGURE 4:**
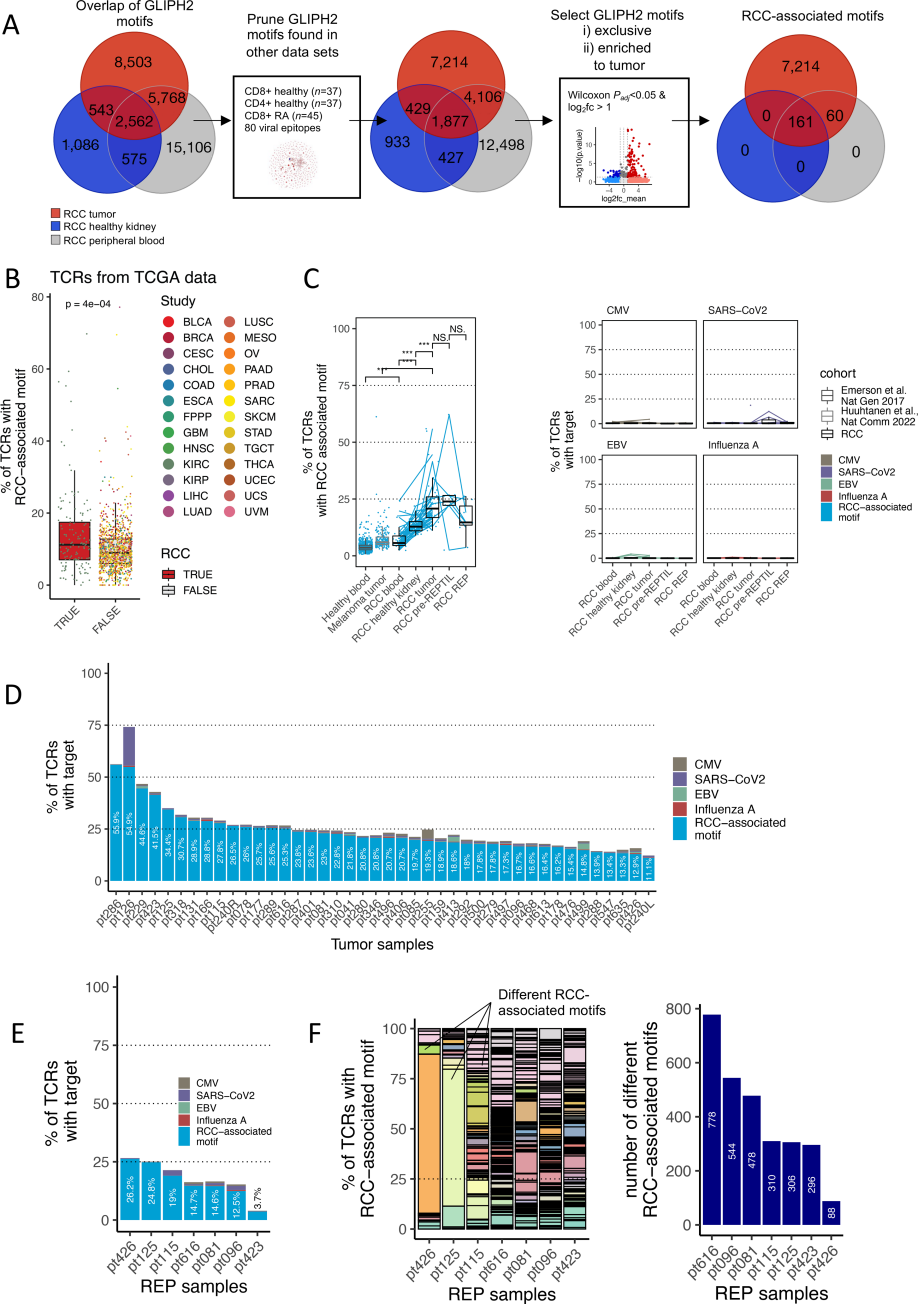
Identification and validation of RCC-associated TCR motifs. **A,** Strategy for the identification of RCC-associated motifs. First, GLIPH2 was separately run on the RCC TCRβ-seq samples: tumor (*n* = 42), healthy kidney (*n* = 24), and PB (*n* = 32) samples. The overlap of the motifs is shown on the Venn diagrams. We subsequently pruned the motifs found in other datasets to increase the likeliness of finding RCC-associated motifs. We retained motifs that were only exclusive to or enriched to the tumor, resulting in a total of 7,435 RCC-associated motifs (Supplementary Table S4). RA = Rheumatoid arthritis. **B,** Validation of RCC-associated TCR motifs. From TCGA cohort (48), we selected samples with at least 100 TCR reads and were left with RCC samples (*n* = 148), as well as samples from 25 different tumors (*n* = 1,042). The *P* value was calculated using the two-sided Wilcoxon test. Tumor sample abbreviations and sample numbers are shown in Supplementary Table S4. Additional validation results are presented in Supplementary Fig. S8. **C,** Proportion of TCRs with RCC-associated motifs in different tissues (left). TCRs with RCC-associated motifs were calculated in samples from our cohort [patients with RCC sampled from PB (*n* = 32), healthy kidney (*n* = 24), tumor (*n* = 42), pre-REP TIL (*n* = 7), REP TIL (*n* = 7), and two comparison cohorts (Emerson and colleagues (46), PB samples (*n* = 786); Huuhtanen and collegaues (47), melanoma biopsy samples (*n* = 216)]. The proportion of anti-viral TCRs was also calculated using TCRGP (ref. 32; right). *P* values were calculated with the Wilcoxon test. Raw data are presented in Supplementary Table S4. **D,** Stacked barplot showing the proportion of TCRs with RCC-associated motifs and anti-viral TCRs in RCC tumors predicted with TCRGP (32). **E,** Stacked barplot showing the proportion of TCRs with RCC-associated motifs and TCRGP (32)-predicted anti-viral TCRs in the REP TIL samples. **F,** The expansion and number of RCC-associated motifs in REP TIL samples. On the left, each colored bar denotes the proportion of individual RCC-associated motifs in each REP TIL sample. The legend for the colors is not shown, as there are 1,790 RCC-associated motifs (please refer to Supplementary Table S4 for all). The right panel shows the total number of different RCC-associated motifs in each REP sample.

After exploring the TCR repertoires between the tumors and the various sample types, we sought to further explore the shared TCR clonotypes using machine learning tools, such as TCRGP (32) and GLIPH2 (42) to discover antigen-specific TCR clusters enriched in the tumor. First, we ran GLIPH2 for each of the pooled tumor (*n* = 42), healthy kidney (*n* = 24), and PB (*n* = 32) TCRβ-seq samples. Similar to previous studies (47), we noticed that although the TCRs were highly conserved between individuals, the antigen-specific motifs were shared between the tumor samples. Next, to prune out the TCRs that were unlikely to target RCC-associated antigens, we filtered out motifs that were also found in the PB samples of healthy donor CD4^+^ (*n* = 37), CD8^+^ (*n* = 37) sorted T cells (34), those from patients with rheumatoid arthritis (ref. 44; *n* = 37), and motifs associated with 80 different viral epitopes from VDJdb (ref. 51; Fig. 4A). As a result, we found 7,214 motifs that were found only in the RCC tumor samples and 221 motifs that were enriched to the tumor samples (*P*_adjusted_ < 0.05, log_2_fc > 1, Benjamini-Hochberg–corrected Wilcoxon test) in comparison with the healthy kidney and/or PB samples. Together, the “tumor-exclusive” and “tumor-enriched” motifs comprised of 7,435 motifs, referred to as RCC-associated motifs henceforth (Supplementary Table S4).

We validated the RCC-associated motifs by calculating their abundance in various TCRβ-seq and scRNA+TCRαβ-seq datasets. In TCGA cohort (48), the proportion of TCRs with RCC-associated motifs was higher in RCC (*n* = 148) than in 25 different cancer types (*n* = 1,042; *P* = 4.0 × 10^−4^, Wilcoxon test; Fig. 4B). Similar observations were made in a cohort of TILs from different scRNA+TCRαβ-seq studies (ref. 49; RCC, *n* = 6; 14 other cancer types, *n* = 76; *P* = 0.05; Supplementary Fig. S12A). In a scRNA+TCRαβ-seq cohort (50) of patients with RCC (*n* = 6), higher levels of RCC-associated TCRs were found in patients who received nivolumab and ipilimumab combination therapy (*n* = 4) than in untreated patients (*n* = 2, *P* value not counted due to low *n*; Supplementary Fig. S12B). The phenotype of the T cells carrying the motifs varied significantly; different motifs were linked to various phenotypes, but mostly to exhausted CD8^+^ T cells, tissue-resident memory T cells, and regulatory T cells (Supplementary Fig. S12C and S12D).

In TCGA cohort of patients with at least 100 recovered TCR reads from the bulk RNA-sequencing data, the number of TCRs (and the overall number of T cells) was associated with worse overall survival (OS) (*P* = 0.033, log-rank test; Supplementary Fig. S12E–S12G). When the number of TCRs with RCC-associated motifs was normalized to the number of TCRs in total, the proportion of TCRs with RCC-associated TCR motifs was not associated with OS (*P* = 0.3, log-rank test between patients with above median proportion of TCRs with RCC motifs compared with those below median proportion of TCRs with RCC motifs; Supplementary Fig. S12H).

### Pre-REP TILs Carry the Most TCRs with RCC-associated Motifs

We further utilized the validated RCC-associated TCR motifs to estimate the proportion of RCC-associated TCRs in our samples using the TCR-epitope recognition machine learning classifier, TCRGP (32). To estimate the frequency of other antigen-specific clones, we predicted the specificity of the TCRs against common endemic viruses such as CMV, EBV, Influenza A, and SARS-CoV-2.

In our RCC samples, the proportion of TCRs with RCC-associated motifs was higher in the PB samples (*n* = 30) than in the healthy donors (ref. 46; *n* = 786, *P* < 0.0001, Wilcoxon test; Fig. 4C, left; Supplementary Table S4). Similarly, our cohort of RCC tumors (*n* = 42) included more TCRs with RCC-associated motifs than the metastatic melanoma patient cohort (ref. 47; *n* = 226 tumor biopsies, *P* < 0.0001; Fig. 4C, left). The proportion of TCRs with RCC-associated motifs was the highest in pre-REP TILs, second in tumors, and third in the REP TILs. Anti-viral TCRs in the pre-REP TIL and REP TIL samples were not enriched (Fig. 4C, right). In addition, we noted a patient (pt240) that underwent two subsequent nephrectomies possessed a higher number of TCRs with RCC-associated motifs in the right kidney (26.5%) than in the left (11.1%), reflective of the different histopathologic tumor (TNM) stages (pT3aNX vs. pT1a; Fig. 4D). The variation in the proportion of TCRs with RCC-associated motifs was higher in the tumor (mean 23.4%, SD 0.1) than in the final REP TIL samples (mean 16.5%, SD 0.08), and the baseline amount of TCRs with RCC-associated motifs was not related to the quantity in the REP TIL products (Fig. 4D and E). The proportion of TCRs with RCC-associated motifs was 4.3–84.4 times greater than that of the anti-viral T cells in the REP TILs, indicating that the anti-viral clonotypes were not enriched in REP TIL samples (Fig. 4E; Supplementary Table S4).

Moreover, the REP TIL samples encompassed a range of different motifs. Notably, the sample with the second highest amount of TCRs with RCC-associated motifs (pt426) had the lowest number of different RCC-associated motifs, as one motif (SAGLAGE*E) occupied most of the repertoire (21.4% of TCRs, 79.3% of TCRs with RCC-associated motifs; Fig. 4F; Supplementary Table S4). Similar results were observed in the patient with the third highest number of TCRs with RCC-associated motifs (pt125, SSGT*GET motif, 17.7% and 68.3%, respectively; Fig. 4F; Supplementary Table S4).

### Single-cell Transcriptomics Reveals Distinct Origins of pre-REP TIL and REP TIL Clonotypes in RCC Tumors

**FIGURE 5:**
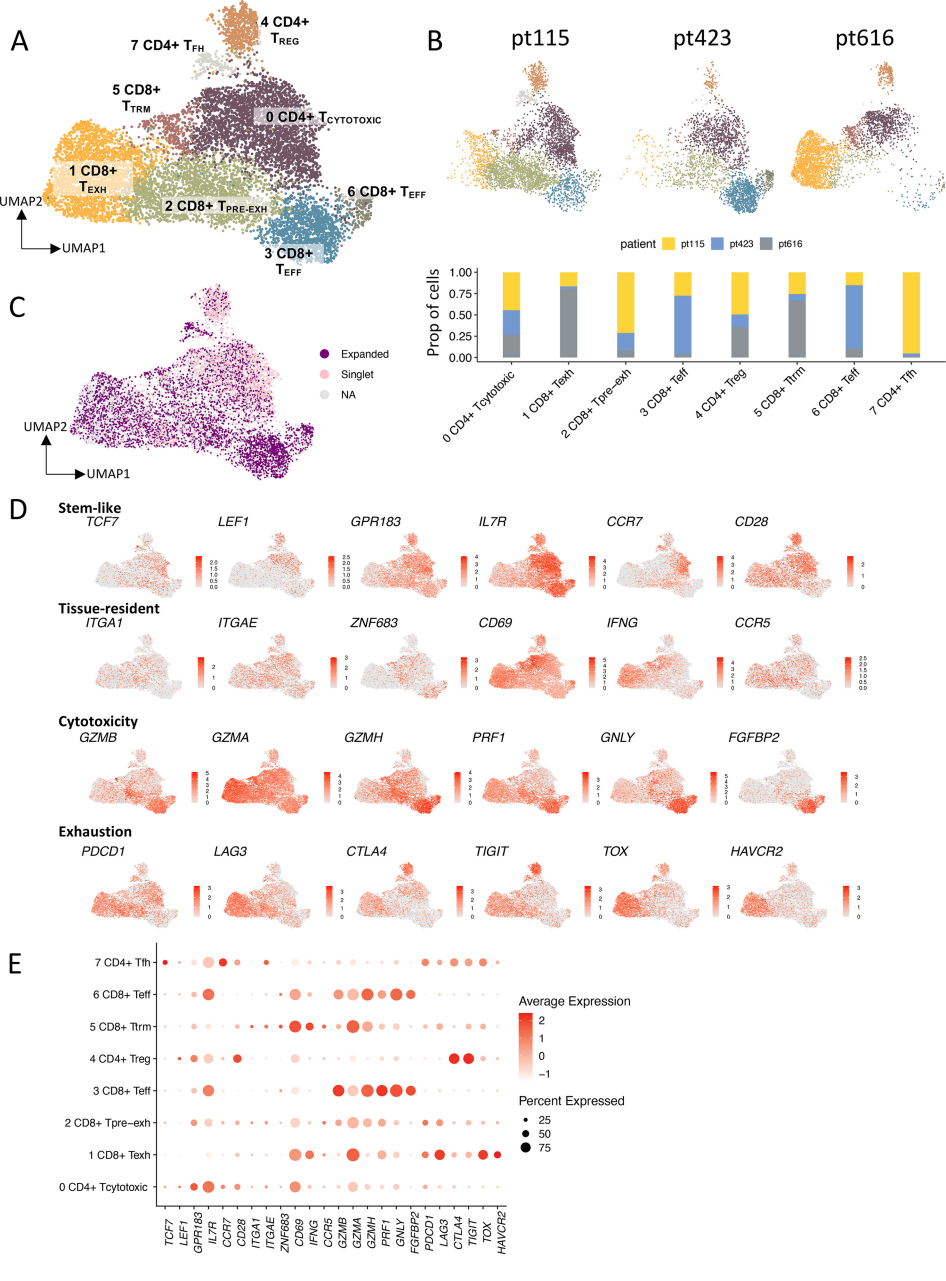
Distinct origins of pre-REP TIL and REP TIL TCR clonotypes in RCC tumors. **A,** Dissociated tumor samples that were enriched for lymphocytes and flow-sorted for CD45^+^ cells (*n* = 3) were analyzed with sc-RNAseq+TCRαβ-seq. The UMAP shows separately clustered T cells, and the UMAP including all cell types can be found in the Supplementary Fig. S9A. Eight different T cell clusters present in the tumor were annotated on the basis of the canonical marker genes, DEGs, and different gene module scores [from Tirosh and colleagues (41)], where each color indicates a distinct T cell cluster. Cluster 0: cytotoxic CD4^+^ T (T_CYTOTOXIC_), cluster 1: exhausted CD8^+^ T cell (T_EXH_), cluster 2: pre-exhausted CD8^+^ T (T_PRE-EXH_), cluster 3: CD8^+^ T effector (T_EFF_), cluster 4: CD4^+^ T_REG_, cluster 5: CD8^+^ tissue resident memory (T_TRM_), cluster 6: CD8^+^ T_EFF_), cluster 7: CD4^+^ T follicular helper (T_FH_). **B,** UMAP showing all clusters present in each patient sample (pt115, pt423, and pt616), with distinct T cell phenotypes. The CD8^+^ T_PRE-EXH_ and CD4^+^ T_FH_ phenotypes were dominant in pt115, pt423 encompassed a more CD8^+^ T_EFF_ and CD4^+^ T_CYTOTOXIC_ phenotype, and pt616 was characterized by CD8^+^ T_EXH_ and CD8^+^ T_TRM_ phenotypes. **C,** Mapped expanded and singlet TCRαβ clonotypes on the tumor UMAP show that the clonotypes lie on distinct parts of the UMAP. Most of the non-expanded (singlet) clonotypes have a CD4^+^ T_CYTOTOXIC_ phenotype (cluster 0), whereas expanded clonotypes are dominant in the CD8^+^ T_EFF_, T_PRE-EXH_ and T_EXH_ phenotypes (cluster 3, 2, and 1, respectively). The relative proportion of the different clonotypes in each of the UMAP clusters is found in Supplementary Fig. S9B. **D,** Scaled expression of selected canonical markers related to stemness, tissue residency, cytotoxicity, and exhaustion in the T cell UMAP representation as in A. **E,** Dot plot showing the scaled average (scale) and percentage (dot size) of expression between the selected DEGs for each UMAP cluster. The full list of the top 10 DEGs and their scaled expressions are found in Supplementary Fig. S9C and S9D).

To further explore the pre-REP TIL and REP TIL clonotypes in greater resolution, paired scRNA+TCRαβ-seq was carried out from lymphocyte-enriched, CD45^+^ flow-sorted dissociated tumor samples (*n* = 3). After removing 5,208 other cells of interest (B cells, monocytes), as well as 4,310 cells representing NK cells (Supplementary Fig. S13A), we filtered out a total of 10,437 T cells. We detected eight different T cell clusters present in the tumor (Fig. 5A), that were annotated on the basis of the canonical marker genes, DEGs, and different gene module scores (ref. 52; Supplementary Fig. S13A; Supplementary Table S5).

Out of the eight clusters, we identified three different CD4^+^ clusters [cluster 0: cytotoxic CD4^+^ T (T_CYTOTOXIC_); cluster 4: CD4^+^ T regulatory (T_REG_); cluster 7: CD4^+^ T follicular helper (T_FH_), and five different CD8^+^ clusters (cluster 1: exhausted CD8^+^ T (T_EXH_); cluster 2: pre-exhausted CD8^+^ T (T_PRE-EXH_); cluster 3: CD8^+^ T effector (T_EFF_); cluster 5: CD8^+^ tissue resident memory (T_TRM_); cluster 6: CD8^+^ T_EFF_]. The annotated clusters were also consistent with those previously described in RCC-related scRNA-seq publications (39, 50, 53, 54). Although all clusters were present in each patient sample, the samples displayed distinct phenotypes. We observed the CD8^+^ T_PRE-EXH_ and CD4^+^ T_FH_ phenotypes in pt115; pt423 encompassed a more CD8^+^ T_EFF_ and CD4^+^ T_CYTOTOXIC_ phenotype; and pt616 was characterized by CD8^+^ T_EXH_ and CD8^+^ T_TRM_ phenotypes (Fig. 5B and C; Supplementary Fig. S13B).

Most of the CD4^+^ T cells were of T_CYTOTOXIC_ phenotype, with high expression of the cytotoxic marker gene, *KLRB1*, as well as tissue resident marker genes (ref. 55; *MYADM*, *ITGAE*, *CD69*; Fig. 5D and E; Supplementary Fig. S13C and S13D). The second largest CD4^+^ T cell cluster corresponded to T_REGS_ (cluster 4), with high expression of T_REG_ marker genes (*FOXP3, IL2RA/CD25, CTLA4, TIGIT*), but also the expression of activation genes (*TNFRSF4/OX40*, *TNFRSF18/GITR*) and *CCR8*, which has been proposed as a marker of clonally expanded T_REGS_ that recognize tumor antigens (56–58). Cluster 7, that was almost exclusive to pt115, showed high expression of the B cell chemoattractant, *CXCL13*, and the expression of other markers related to the T_FH_ phenotype (*CXCR5*, *CCR7, PDCD1*), with no *FOXP3* expression, making them plausible T_FH_ cells (Fig. 5D and E; Supplementary Fig. S13C and S13D).

The CD8^+^ T cells included two different T_EFF_ populations (cluster 3 and 6), with high expression of cytotoxicity genes (*GZMA/B/M/H, PRF1, GNLY*) and NK-related receptors (*FCGR3A/CD16*, different killer immunoglobulin-like receptors (KIRs); Fig. 5D and E; Supplementary Fig. S13C and S13D). In addition, we identified a CD8^+^ T_TRM_ cluster (cluster 5) with the expression of T_TRM_ markers (*ITGA1, ITGAE, and CD69)*. Furthermore, the T_PRE-EXH_ (cluster 2) had the highest expression of *TCF7* (encoding TCF1) out of CD8^+^ T cells, along with some other markers related to stem-like properties (refs. 9, 59–64; *LEF1*, *GPR183*, *CCR7*; Fig. 5D and E; Supplementary Fig. S13C and S13D). This cluster was named pre-exhausted as in other publications, due to its expression of exhaustion-related markers such as *PDCD1* (encoding PD-1), but the lack of expression of other immune checkpoint molecules associated with terminal exhaustion, such as *HAVCR2* (encoding TIM-3) and *TOX* (65–70). These terminal exhaustion markers were expressed by cluster 1 (Fig. 5D and E; Supplementary Fig. S13C and S13D). On the basis of the TCR-repertoire clonality analysis, the terminally exhausted cluster 1 had the most clonally restricted population (Gini index 0.8), making it plausible that the T_PRE-EXH_ cluster 2 indeed had stem-like properties, and thus gave rise to the cells in the terminally exhausted cluster 1 (Supplementary Fig. S14A and S14B).

**FIGURE 6:**
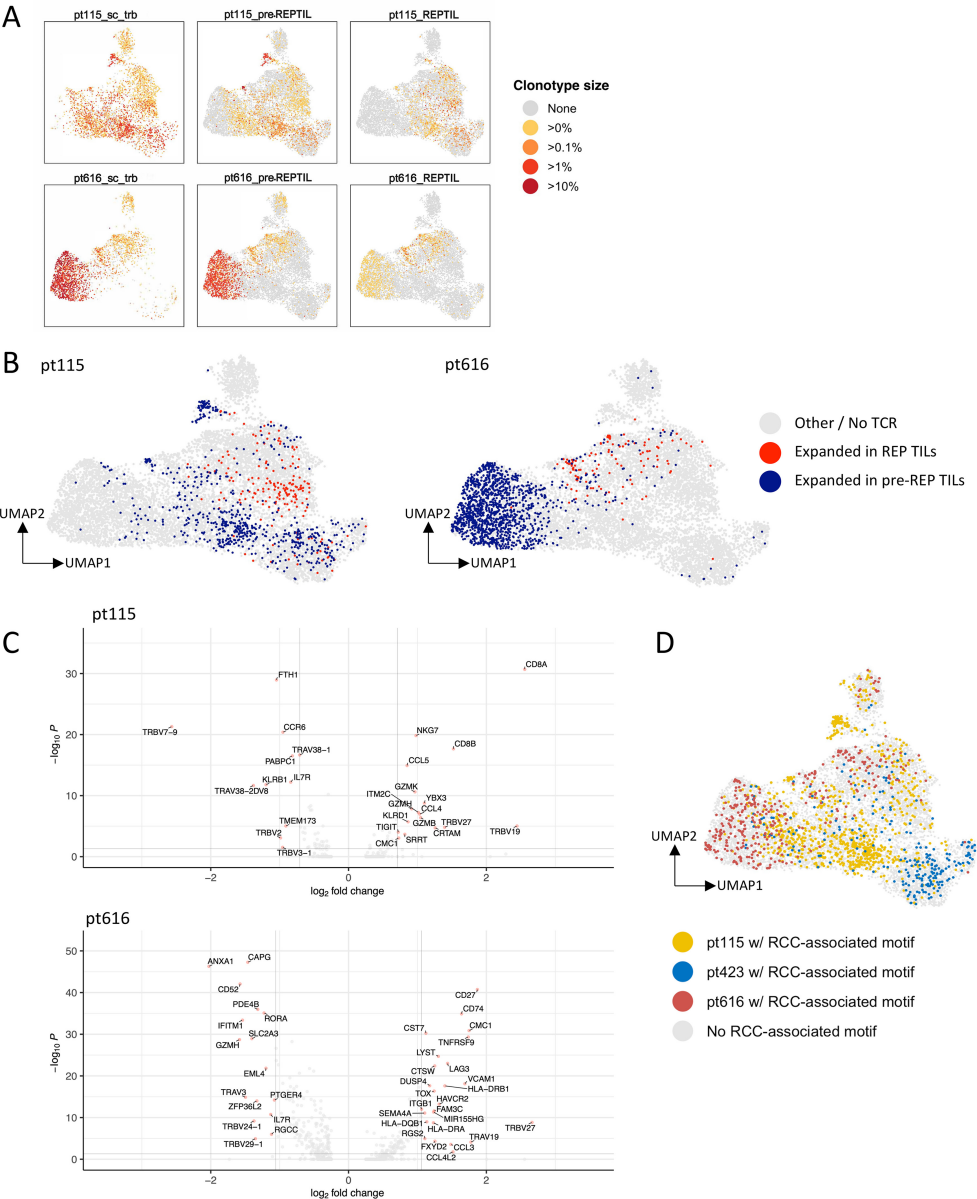
Expanded clonotypes between the pre-REP TILs and REP TILs show distinct phenotypic differences within the tumor. **A,** UMAPs comparing the size of the T cell clonotypes found in the sc-RNAseq+TCRαβ-seq and TCRβ-seq data from 2 patients (pt115 and pt616). Each dot represents a clone in which the color corresponds to the size of the clone in the sc-RNAseq+TCRαβ-seq (left column), TCRβ-seq data from the pre-REP TILs (middle column), and TCRβ-seq data from the REP TILs (right column). Representations for all samples, including the available tumor and healthy kidney samples are found in Supplementary Fig. S10C). **B,** UMAPs showing the expanded pre-REP TIL and REP TIL clones in pt115 and pt616 (Benjamini-Hochberg–corrected two-sided Fisher’s exact test, *P*_adjusted_ < 0.05). In both patients, more pre-REP TIL clonotypes were expanded in the CD8^+^ T_PRE-EXH_ (pt115) and CD8^+^ T_EXH_ (pt616) phenotypes, whereas expanded REP TIL clonotypes were mainly characterized by the CD4^+^ T_CYTOTOXIC_ cell phenotype. **C,** Volcano plots showing the DEGs in the expanded REP TIL samples (right) compared with those in the pre-REP TILs (left) in pt115 and pt616. In pt115, genes that were most upregulated (right) in the expanded REP TIL clonotypes were *NKG7*, *CCL5,* and *CD8A*, together with those related to cytotoxicity, such as *GZMB*, *GZMH*, and *GZMK*. In pt616, genes related to activation such as *HLA-DRB1*, *HLA-DRA*, chemokines such as *CCL3* and *CCL4L2*, as well as *LAG-3* were upregulated in the expanded REP TIL clonotypes. In both patients, *IL7R* was among the DEGs in the expanded pre-REP TIL clonotypes. **D,** UMAP showing T cells carrying the RCC-associated motifs found in the sc-RNAseq+TCRαβ-seq tumor patients (*n* = 3). T cells carrying the RCC-associated motifs showed distinct T cell phenotypes in each individual sample.

We also analyzed the size of the clonotypes in the scRNA+TCRαβ-seq and TCRβ-seq data. The largest clonotypes found in the scRNA+TCRαβ-seq data were mainly found in the TCRβ-seq pre-REP TILs, whereas the REP TILs encompassed smaller clonotypes in pt115 and pt616 (Fig. 6A). We further analyzed the size of the clonotypes in the tumor scRNA+TCRαβ-seq data for pt115 and pt616, as well as from the TCRβ-seq data in the corresponding samples (healthy, tumor, pre-REP TIL, and REP TIL) and found that the expanded clonotypes sequestered to distinct parts of the UMAP (Fig. 6B; Supplementary Fig. S14C). In the third patient (pt423), we did not find matching clones between the tumor scRNA+TCRαβ-seq, pre-REP TIL and REP TIL samples (Supplementary Fig. S14C), suggesting the result of either sampling bias, or that the dissociated tumor cells and REP TILs were derived from physically distant regions of the tumor tissue.

Subsequently, we visualized the shared and expanding clonotypes between the pre-REP TIL and REP TIL samples as well as their corresponding phenotypes. We defined the statistically significant expanded clonotypes as those at least ≥0.1% in either one of the pre-REP TIL or REP TIL samples, using Benjamini-Hochberg–corrected two-sided Fisher’s exact test. In both patients, the largest clonotypes in the pre-REP TILs resulted from large tumor-infiltrating CD8^+^ T cell clones (Fig. 6B). However, the CD8^+^ T cell phenotypes in the patients were heterogeneous. In pt115, the expanded pre-REP TIL clonotypes were more of the CD8^+^ T_PRE-EXH_ phenotype (cluster 2), whereas in pt616, most of the expanded pre-REP TIL clonotypes were of the CD8^+^ T_EXH_ (cluster 1) phenotype (Figs. 5A and 6B). The clonotypes that were expanded in the REP TIL samples displayed a CD4^+^ T_CYTOTOXIC_ phenotype (cluster 0) from the original tumor sample for both cases (Figs. 5A and 6B).

Next, we analyzed the DEGs between the statistically significant clonotypes expanded in the REP TILs for pt115 and pt616. In pt115, the genes that were most upregulated in the expanded REP TIL clonotypes in the original tumor were *NKG7*, *CCL5,* and *CD8A*, but also those related to cytotoxicity, such as *GZMB/H/K* (Fig. 6C). In pt616, genes related to activation such as *HLA-DRB1*, *HLA-DRA*, chemokines such as *CCL3* and *CCL4L2*, as well as *LAG-3* were observed, in line with our previous observations (Fig. 6C). Furthermore, gene ontology (GO) pathway analysis using clusterProfiler (71) showed T cell activation and response to IFNγ among the top GO pathways enriched in pt115 pre-REP TIL clonotypes in the original tumor, whereas pathways related to cytokine production and T cell differentiation were found in the original REP TIL clonotypes (Supplementary Fig. S15A). In pt616, IFNγ signaling, T cell activation, and regulation were among the most enriched pathways in the pre-REP TIL clonotypes, whereas cellular pathways related to cotranslational protein targeting were enriched in the REP TIL clonotypes (Supplementary Fig. S15B).

Finally, we explored the phenotype of T cells with RCC-associated motifs found in the three tumor samples (pt115, pt423, pt616) and observed that the phenotypes of the T cells with RCC-associated motifs were largely distinct (Fig. 6D). T cells with RCC-associated motifs in pt115 were concentrated in the CD4^+^ T_FH_ compartment (cluster 7), pt423 displayed a CD8^+^ T_EXH_ cell phenotype (cluster 1), and pt616 displayed the CD8^+^ T_PRE-EXH_ phenotype (cluster 2; Fig. 6D; Supplementary Fig. S15C). The same clonotypes that carried RCC-associated motifs were also observed in the pre-REP TIL and REP TIL products for pt115 and pt616 and were among the most expanded clonotypes (Fig. 6B and D). Of note, all the patients were in remission during the study follow-up after the surgery, thus the phenotypes could not be linked to clinical outcomes.

## Discussion

Adoptive TIL therapies have been successfully used in melanoma patients, even in anti-PD-1 therapy refractory cases (16, 18); thus, the interest in applying TILs to other tumor types has increased. Previous studies have demonstrated that TILs can be expanded from RCC tumor tissues (20, 72–75). However, the composition, possible tumor reactivity, and clinical efficacy of RCC TILs remain unclear. Some studies have shown that the infiltration of T cells is associated with good prognosis in RCC (7–9), whereas others have reported contradictory results in which T cell infiltration has been associated with poor prognosis (4–6, 76). In addition, although the REP TILs are mostly used as the final infusion product in the adoptive TIL therapies, there are only a few studies that have explored the differences in the phenotype between the original, minimally cultured pre-REP, and REP TILs (27, 77, 78). Therefore, we performed in-depth analyses of the pre-REP TILs and REP TILs that were expanded using a clinical expansion protocol from patients with treatment-naïve RCC. We also compared the TIL phenotype, function, and TCR repertoires with those originating from the autologous tumor tissue to shed light on the changes that occur during the *ex vivo* culture conditions. Our results show that the immunophenotype and TCR repertoire drastically change between the tumors, pre-REP TILs, and REP TILs, suggesting that initially, only a proportion of T cells expand from the tumor tissue (pre-REP TILs). In addition, from the pre-REP TILs, a smaller proportion of T cells further expand during the REP protocol.

Phenotypically, the REP TILs displayed a CD4^+^ T_CYTOTOXIC_-cell phenotype, which was drastically different from the T cells found in the TME, where a significant proportion of T cells exhibited CD8^+^ T_PRE-EXH_ and T_EXH_ phenotypes. The elevated expression of PD-1 in the tumor CD4^+^ and CD8^+^ T cells indicates the exhausted nature of T cells in the TME, which may ultimately hinder their expansion. These exhausted PD-1–expressing CD8^+^ T cells have traditionally been known as tumor-reactive and targets of ICI therapies. The exhausted clonotypes were usually large, and by comparing the TCR repertoires of different sample types, we discovered that the tumor and pre-REP TIL TCR repertoires overlapped, whereas the REP TILs possessed distinct repertoires. The most abundant clonotypes in the REP TILs were observed to be small, and/or unique in the pre-REP TIL and tumor samples, suggesting that many of the larger clonotypes present in the tumor and pre-REP TILs did not proliferate during the REP protocol. Accordingly, the scRNA+TCRαβ-seq data confirmed that the expanded pre-REP TIL clonotypes mostly encompassed a CD8^+^ T_EXH_-cell phenotype in the original tumor samples, whereas the most expanded REP TIL clonotypes originate from tumor CD4^+^ T_CYTOTOXIC_ cells.

Many previous studies have not been able to assess the proportion of tumor-reactive or bystander (such as anti-viral) T cell clonotypes following the REP expansion. In contrast to metastatic melanoma (47), there is not yet a database of epitope-specific TCRs against RCC-associated antigens. However, with modern bioinformatics tools that assess antigen specificities based on TCR similarities (42), we were able to cluster TCRs with shared motifs into putative antigen-specific groups. We discovered RCC-associated TCR motifs and were able to validate them in multiple TCRβ-seq and scRNA+TCRαβ-seq datasets, confirming their enrichment specifically in RCC tumors and not in the healthy or other cancer types. We further utilized the RCC-associated motifs to estimate the proportion of tumor-reactive TCRs, which were 4.3–84.4 times greater than the anti-viral (CMV, Influenza A, EBV, SARS-CoV2) TCR targets. Our analysis reiterates that REP TILs are specific to targets other than the large, exhausted clones, and without clinical follow-up, it remains unknown whether the expanded TCRs in the REP TIL products are optimal for efficient tumor killing.

Studies on pancreatic ductal adenocarcinoma (PDA) tumors have also shown that the TCR repertoires in TILs vastly change during *in vitro* expansions, with the loss of tumor-dominant T cell clones and emergence of new T cell clones that are barely detectable in the tumor (79). These changes are thought to be driven by differences in the *in vitro* expansion capacity of the T cell clones. In our RCC samples, the expansion potential was inversely correlated with the CD4^+^ T cell phenotype in the tumor. In PDA, the heterogeneity of the TILs resulted in TCR repertoires that were greatly divergent between TIL cultures derived from distant tumor samples of the same patient, suggesting that culture-induced changes in clonal composition are likely to affect the tumor reactivity of TIL preparation (79).

Further studies are necessary to explore how to improve TIL expansion protocols, and whether TIL-based therapies for other T cell–rich tumors in addition to melanoma, will be effective. When tumor-targeting T cells are better recognized, it would be possible to selectively isolate the cytotoxic or tumor-reactive CD8^+^ T cells, and expand them separately, such as with autologous T cells, to increase the tumor killing potential. In addition, although not analyzed in our study, the use of NK and NKT cells as well as other immune cell types may hold potential for combinatorial use with TILs. In conclusion, our study highlights the differences between pre-REP TILs and REP TILs from primary RCC tumor samples and provides tools to analyze RCC-associated T cells in other datasets, such as during ICI therapies.

## Supplementary Material

Table S1 Supplementary Table S1 shows the RCC patient data including clinical parameters and status

Table S2 Supplementary Table S2 shows the list of all markers, antibodies and their catalog numbers used for multi-parameter flow cytometry immunophenotyping, co-culture and T-cell activation assays.

Table S3 Supplementary Table S3 shows the sample availability, usage and TIL expansion potentials.

Table S4 Supplementary Table S4 shows the publicly validated RCC-associated motifs, together with their antigen-specificities with TCRGP, and motifs found in the REP TILs using GLIPH2.

Table S5 Supplementary Table S5 shows the canonical marker genes, differentially expressed genes (DEGs), and gene modules scores used to annotate the T-cell UMAP clusters

Figure S1 Figure S1 shows a schematic of the TIL expansion protocol.

Figure S2 Figure S2 shows representative flow gating strategies for the immunophenotyping of various sample types (tumor, healthy kidney, pre-REP TILs and REP TILs), as well as the co-culture assays.

Figure S3 Figure S3 shows representative gating strategies for the immune subset populations and T-cell subtypes for the healthy kidney, tumor, pre-REP TIL and REP TIL samples.

Figure S4 Figure S4 shows representative gating strategies for the different marker expressions in the CD4+ and CD8+ T-cells for the different samples (healthy kidney, tumor, pre-REP TIL and REP TIL).

Figure S5 Figure S5 shows representative gating strategies for the co-culture assays with different timepoints (6h, 48h) and conditions (baseline, unstimulated, T-cell stimulated).

Figure S6 Figure S6 shows the flow cytometric analyses of the immune cell subsets, T-cell phenotypes, and marker expressions, as well as correlation analyses between the T-cell subsets and the expansion potential (fold-change of REP TILs/pre-REP TILs).

Figure S7 Figure S7 shows analyses of the co-culture assays involving CD107a/b, IFN-y,TNF-a and GzB expressions.

Figure S8 Figure S8 shows the analyses of the different clonotype sizes and tracking their abundancies found in the bulk TCRb-seq data.

Figure S9 Figure S9 shows the correlation of overlapping clonotypes between the REP TILs and pre-REP TILs.

Figure S10 Figure S10 shows correlation of overlapping clonotypes between the tumor and pre-REP TILs.

Figure S11 Figure S11 shows treemaps of various clonotypes found in the patient samples and the matches to viral-specific TCRs.

Figure S12 Figure S12 shows the validation of RCC-associated TCR motifs in various cohorts.

Figure S13 Figure S13 shows analyses from the scRNA+TCRab-seq data, including the different UMAP clusters, differentially expressed genes and canonical marker gene expressions.

Figure S14 Figure S14 shows the clonalities, expression scores and size of clonotypes represented in the T-cell UMAP clusters.

Figure S15 Figure S15 shows pathway enrichment analyses for the pre-REP TILs and REP TILs, as well as the abundance of the RCC-associated motifs found in the UMAP T-cell clusters for each tumor sample.
