# Supplementary material for: Immunologic Characterization and T cell Receptor Repertoires of Expanded Tumor-infiltrating Lymphocytes in Patients with Renal Cell Carcinoma
Source: Cancer Res Commun. 2023 Jul 18;3(7):1260–76. doi: 10.1158/2767-9764.CRC-22-0514 (PMC10361538; doi:10.1158/2767-9764.CRC-22-0514)
Supplement: Figure S1 — shows a schematic of the TIL expansion protocol. [file crc-22-0514-s06.pptx]

## Slide 1
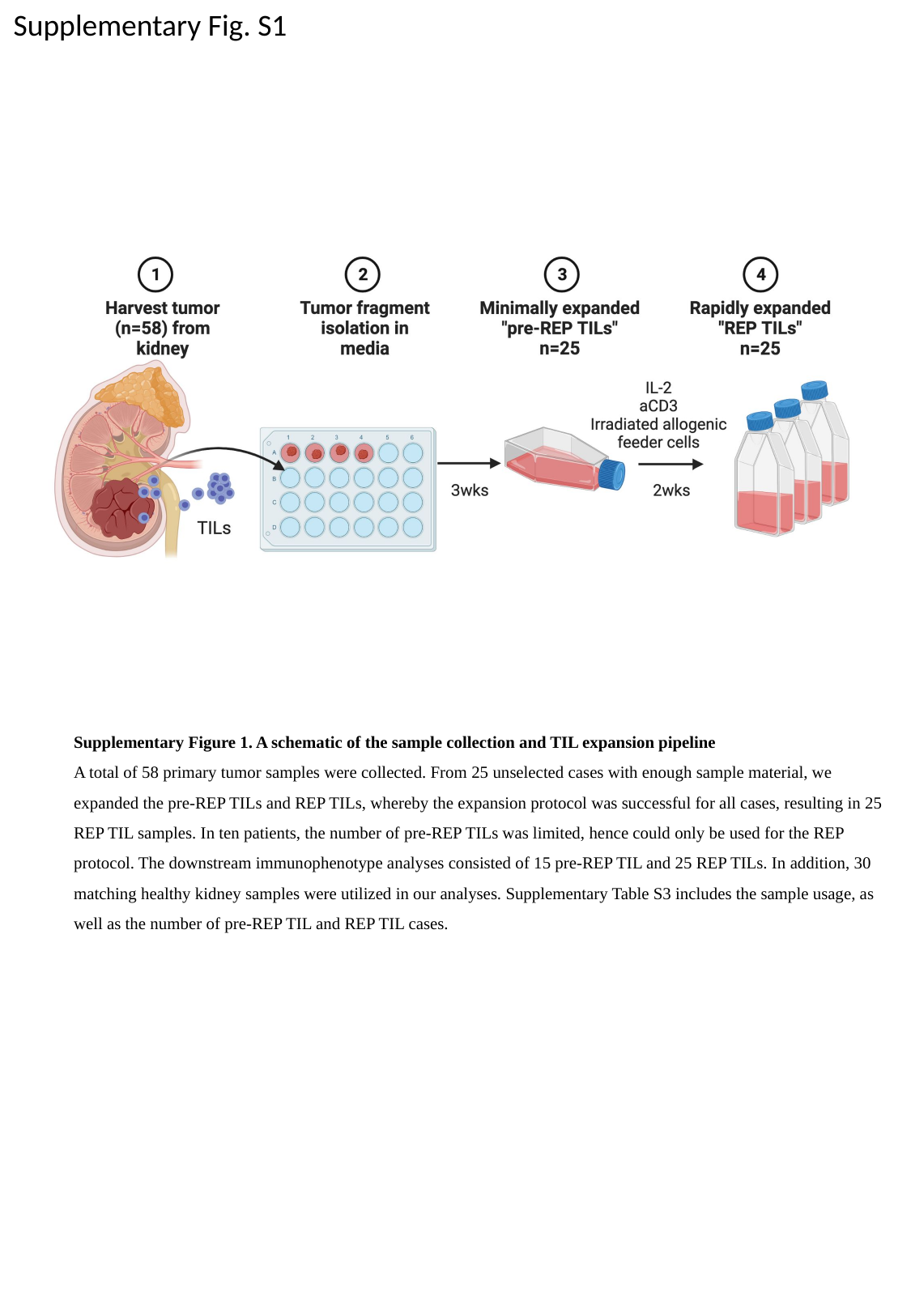

Supplementary Fig. S1
Supplementary Figure 1. A schematic of the sample collection and TIL expansion pipeline
A total of 58 primary tumor samples were collected. From 25 unselected cases with enough sample material, we expanded the pre-REP TILs and REP TILs, whereby the expansion protocol was successful for all cases, resulting in 25 REP TIL samples. In ten patients, the number of pre-REP TILs was limited, hence could only be used for the REP protocol. The downstream immunophenotype analyses consisted of 15 pre-REP TIL and 25 REP TILs. In addition, 30 matching healthy kidney samples were utilized in our analyses. Supplementary Table S3 includes the sample usage, as well as the number of pre-REP TIL and REP TIL cases.
