# Supplementary material for: Immunologic Characterization and T cell Receptor Repertoires of Expanded Tumor-infiltrating Lymphocytes in Patients with Renal Cell Carcinoma
Source: Cancer Res Commun. 2023 Jul 18;3(7):1260–76. doi: 10.1158/2767-9764.CRC-22-0514 (PMC10361538; doi:10.1158/2767-9764.CRC-22-0514)
Supplement: Figure S2 — shows representative flow gating strategies for the immunophenotyping of various sample types (tumor, healthy kidney, pre-REP TILs and REP TILs), as well as the co-culture assays. [file crc-22-0514-s07.pptx]

## Slide 1
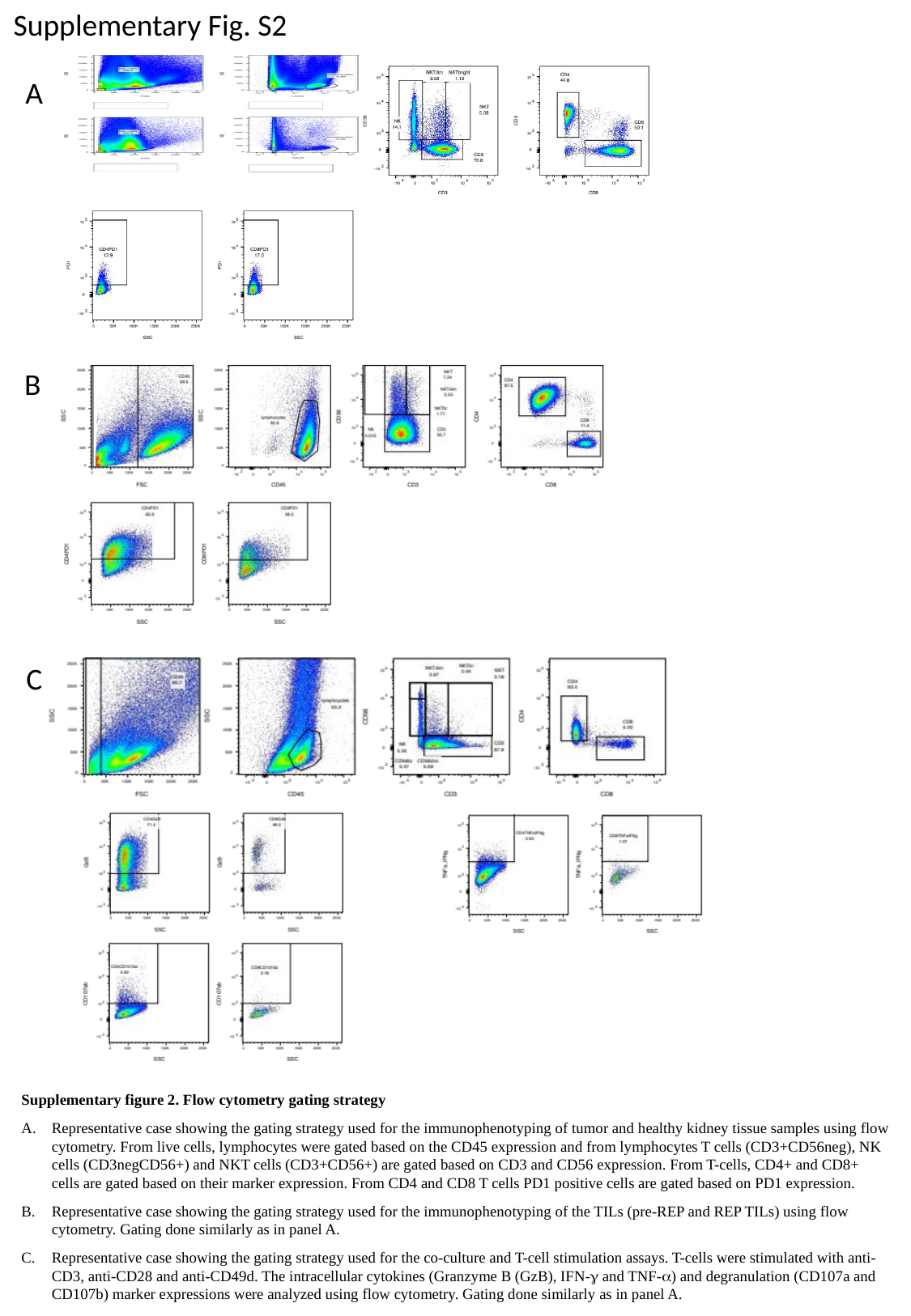

Supplementary Fig. S2
A
B
C
Supplementary figure 2. Flow cytometry gating strategy
Representative case showing the gating strategy used for the immunophenotyping of tumor and healthy kidney tissue samples using flow cytometry. From live cells, lymphocytes were gated based on the CD45 expression and from lymphocytes T cells (CD3+CD56neg), NK cells (CD3negCD56+) and NKT cells (CD3+CD56+) are gated based on CD3 and CD56 expression. From T-cells, CD4+ and CD8+ cells are gated based on their marker expression. From CD4 and CD8 T cells PD1 positive cells are gated based on PD1 expression.
Representative case showing the gating strategy used for the immunophenotyping of the TILs (pre-REP and REP TILs) using flow cytometry. Gating done similarly as in panel A.
Representative case showing the gating strategy used for the co-culture and T-cell stimulation assays. T-cells were stimulated with anti-CD3, anti-CD28 and anti-CD49d. The intracellular cytokines (Granzyme B (GzB), IFN-γ and TNF-α) and degranulation (CD107a and CD107b) marker expressions were analyzed using flow cytometry. Gating done similarly as in panel A.
