# Supplementary material for: Immunologic Characterization and T cell Receptor Repertoires of Expanded Tumor-infiltrating Lymphocytes in Patients with Renal Cell Carcinoma
Source: Cancer Res Commun. 2023 Jul 18;3(7):1260–76. doi: 10.1158/2767-9764.CRC-22-0514 (PMC10361538; doi:10.1158/2767-9764.CRC-22-0514)
Supplement: Figure S3 — shows representative gating strategies for the immune subset populations and T-cell subtypes for the healthy kidney, tumor, pre-REP TIL and REP TIL samples. [file crc-22-0514-s08.pptx]

## Slide 1
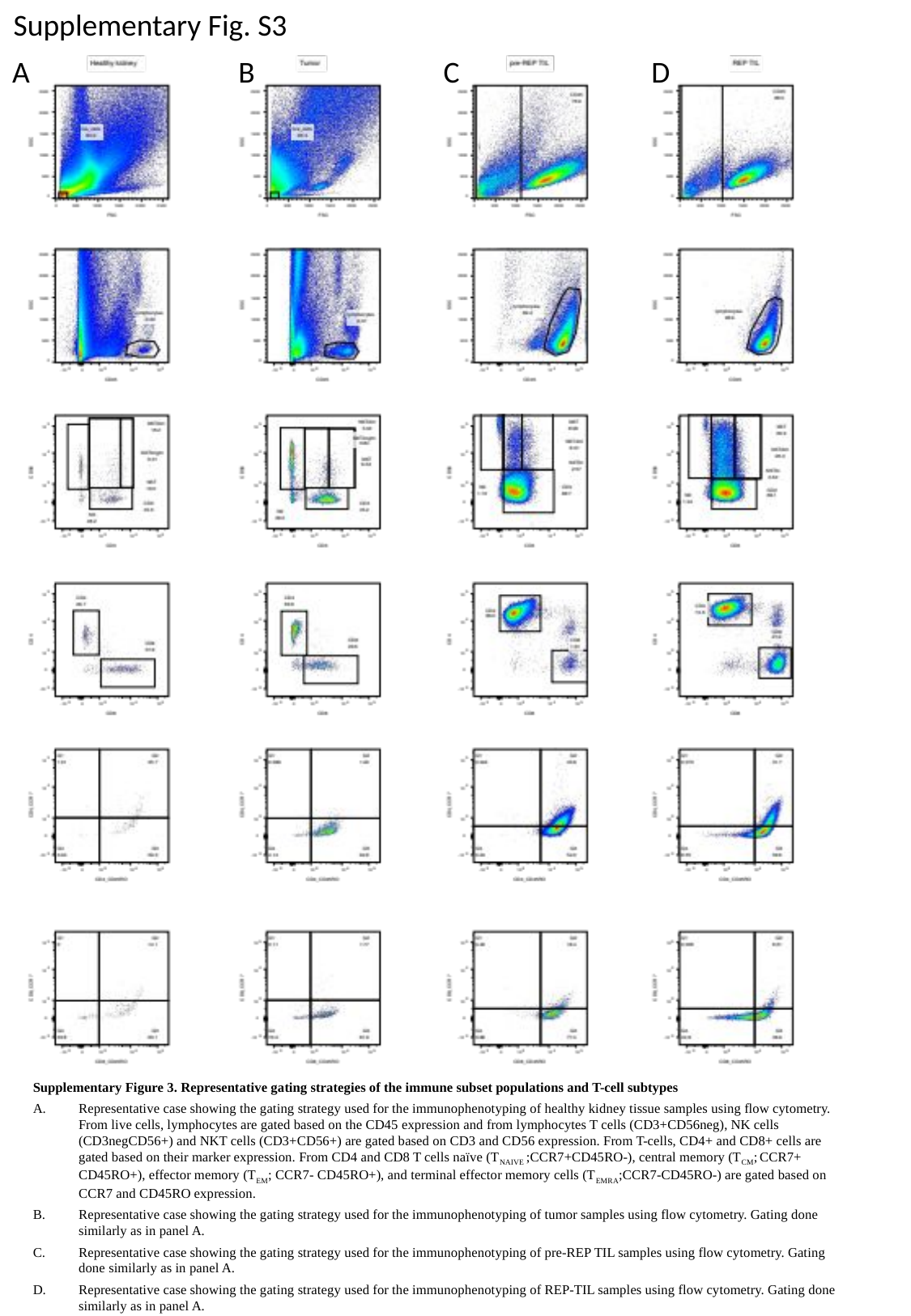

Supplementary Fig. S3
A
B
C
D
Supplementary Figure 3. Representative gating strategies of the immune subset populations and T-cell subtypes
Representative case showing the gating strategy used for the immunophenotyping of healthy kidney tissue samples using flow cytometry. From live cells, lymphocytes are gated based on the CD45 expression and from lymphocytes T cells (CD3+CD56neg), NK cells (CD3negCD56+) and NKT cells (CD3+CD56+) are gated based on CD3 and CD56 expression. From T-cells, CD4+ and CD8+ cells are gated based on their marker expression. From CD4 and CD8 T cells naïve (TNAIVE ;CCR7+CD45RO-), central memory (TCM; CCR7+ CD45RO+), effector memory (TEM; CCR7- CD45RO+), and terminal effector memory cells (TEMRA;CCR7-CD45RO-) are gated based on CCR7 and CD45RO expression.
Representative case showing the gating strategy used for the immunophenotyping of tumor samples using flow cytometry. Gating done similarly as in panel A.
Representative case showing the gating strategy used for the immunophenotyping of pre-REP TIL samples using flow cytometry. Gating done similarly as in panel A.
Representative case showing the gating strategy used for the immunophenotyping of REP-TIL samples using flow cytometry. Gating done similarly as in panel A.
