# Supplementary material for: Immunologic Characterization and T cell Receptor Repertoires of Expanded Tumor-infiltrating Lymphocytes in Patients with Renal Cell Carcinoma
Source: Cancer Res Commun. 2023 Jul 18;3(7):1260–76. doi: 10.1158/2767-9764.CRC-22-0514 (PMC10361538; doi:10.1158/2767-9764.CRC-22-0514)
Supplement: Figure S4 — shows representative gating strategies for the different marker expressions in the CD4+ and CD8+ T-cells for the different samples (healthy kidney, tumor, pre-REP TIL and REP TIL). [file crc-22-0514-s09.pptx]

## Slide 1
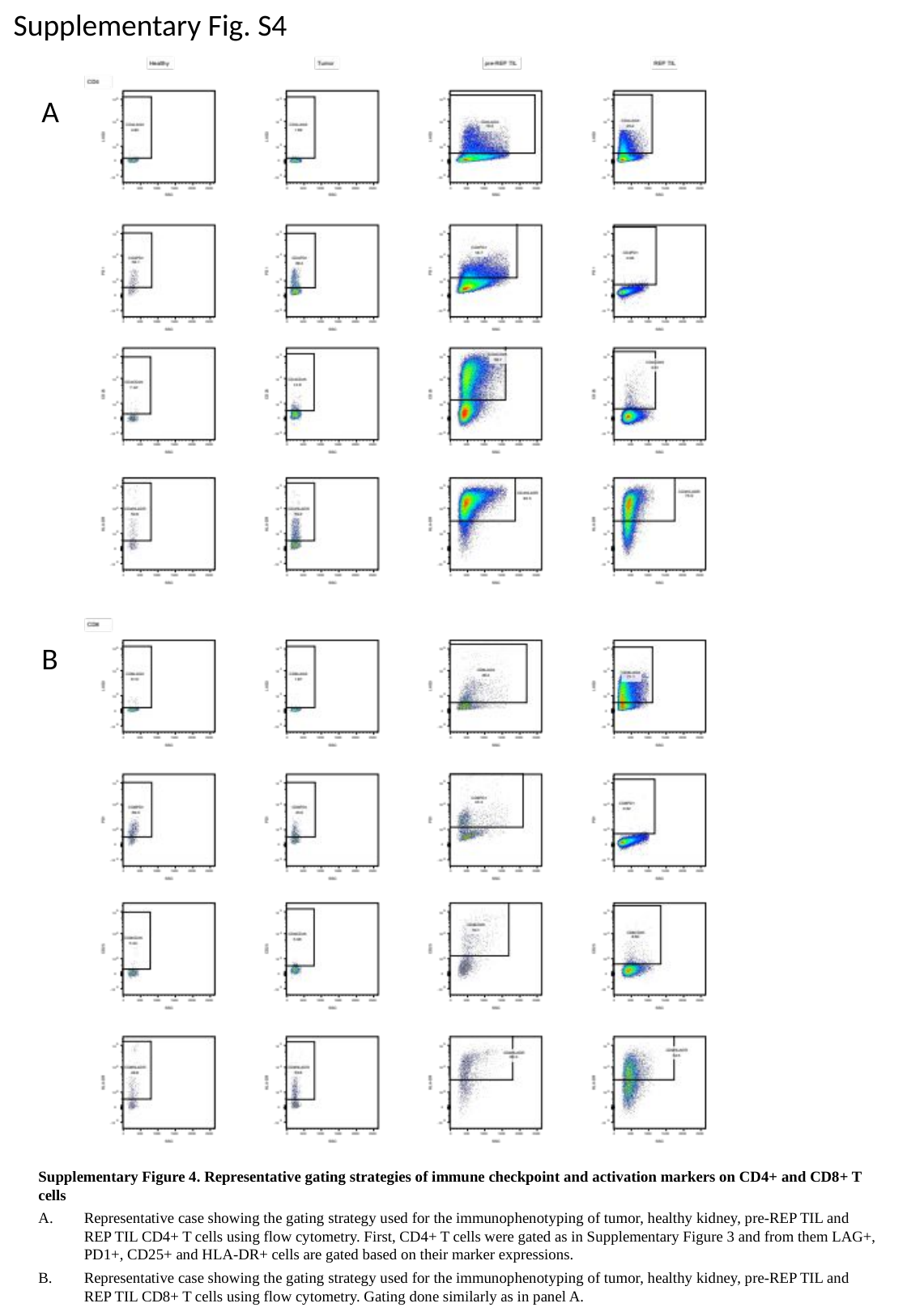

Supplementary Fig. S4
A
B
Supplementary Figure 4. Representative gating strategies of immune checkpoint and activation markers on CD4+ and CD8+ T cells
Representative case showing the gating strategy used for the immunophenotyping of tumor, healthy kidney, pre-REP TIL and REP TIL CD4+ T cells using flow cytometry. First, CD4+ T cells were gated as in Supplementary Figure 3 and from them LAG+, PD1+, CD25+ and HLA-DR+ cells are gated based on their marker expressions.
Representative case showing the gating strategy used for the immunophenotyping of tumor, healthy kidney, pre-REP TIL and REP TIL CD8+ T cells using flow cytometry. Gating done similarly as in panel A.
