# Supplementary material for: Immunologic Characterization and T cell Receptor Repertoires of Expanded Tumor-infiltrating Lymphocytes in Patients with Renal Cell Carcinoma
Source: Cancer Res Commun. 2023 Jul 18;3(7):1260–76. doi: 10.1158/2767-9764.CRC-22-0514 (PMC10361538; doi:10.1158/2767-9764.CRC-22-0514)
Supplement: Figure S5 — shows representative gating strategies for the co-culture assays with different timepoints (6h, 48h) and conditions (baseline, unstimulated, T-cell stimulated). [file crc-22-0514-s10.pptx]

## Slide 1
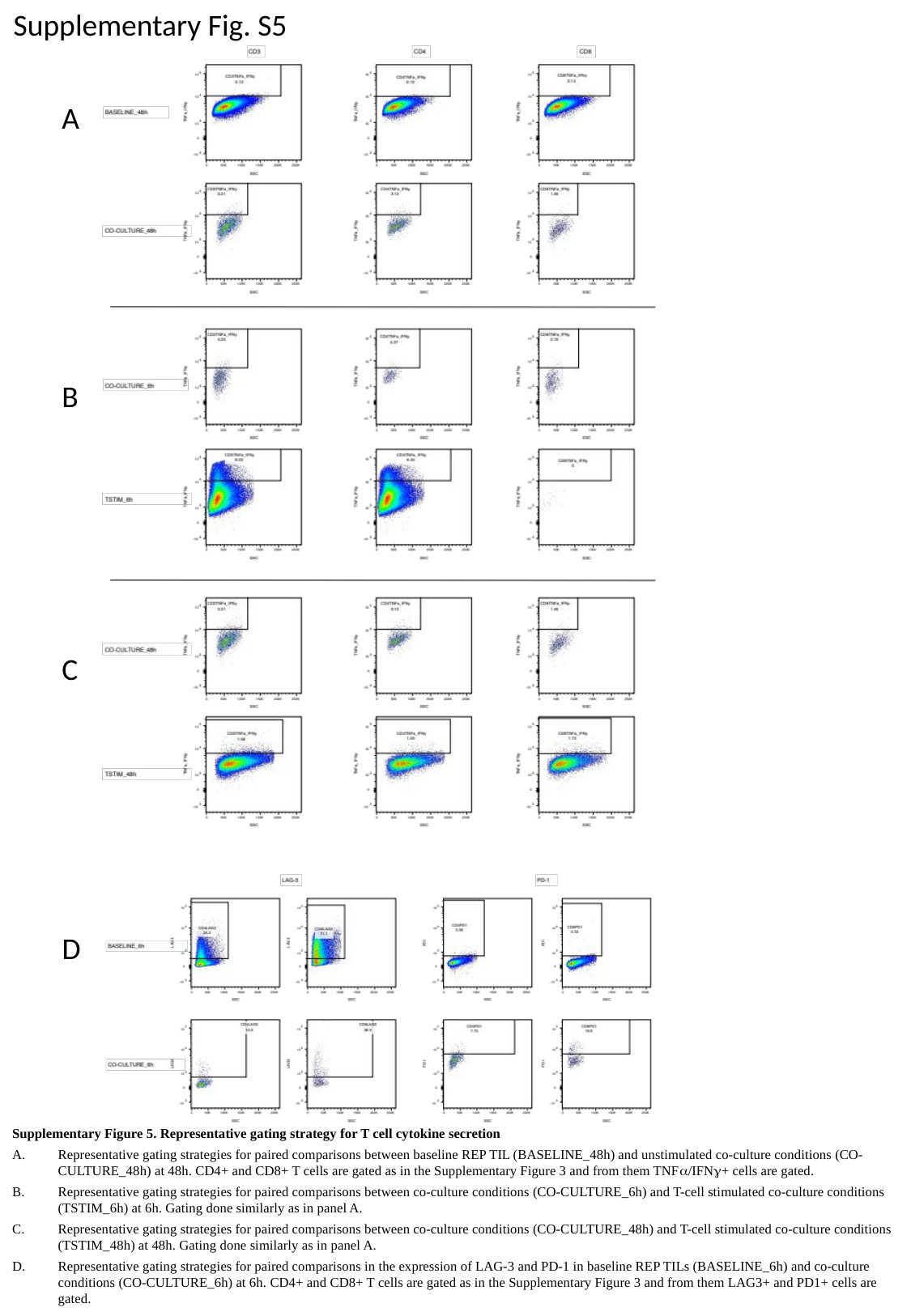

Supplementary Fig. S5
A
B
C
D
Supplementary Figure 5. Representative gating strategy for T cell cytokine secretion
Representative gating strategies for paired comparisons between baseline REP TIL (BASELINE_48h) and unstimulated co-culture conditions (CO-CULTURE_48h) at 48h. CD4+ and CD8+ T cells are gated as in the Supplementary Figure 3 and from them TNFa/IFNg+ cells are gated.
Representative gating strategies for paired comparisons between co-culture conditions (CO-CULTURE_6h) and T-cell stimulated co-culture conditions (TSTIM_6h) at 6h. Gating done similarly as in panel A.
Representative gating strategies for paired comparisons between co-culture conditions (CO-CULTURE_48h) and T-cell stimulated co-culture conditions (TSTIM_48h) at 48h. Gating done similarly as in panel A.
Representative gating strategies for paired comparisons in the expression of LAG-3 and PD-1 in baseline REP TILs (BASELINE_6h) and co-culture conditions (CO-CULTURE_6h) at 6h. CD4+ and CD8+ T cells are gated as in the Supplementary Figure 3 and from them LAG3+ and PD1+ cells are gated.
