# Supplementary material for: Immunologic Characterization and T cell Receptor Repertoires of Expanded Tumor-infiltrating Lymphocytes in Patients with Renal Cell Carcinoma
Source: Cancer Res Commun. 2023 Jul 18;3(7):1260–76. doi: 10.1158/2767-9764.CRC-22-0514 (PMC10361538; doi:10.1158/2767-9764.CRC-22-0514)
Supplement: Figure S6 — shows the flow cytometric analyses of the immune cell subsets, T-cell phenotypes, and marker expressions, as well as correlation analyses between the T-cell subsets and the expansion potential (fold-change of REP TILs/pre-REP TILs). [file crc-22-0514-s11.pptx]

## Slide 1
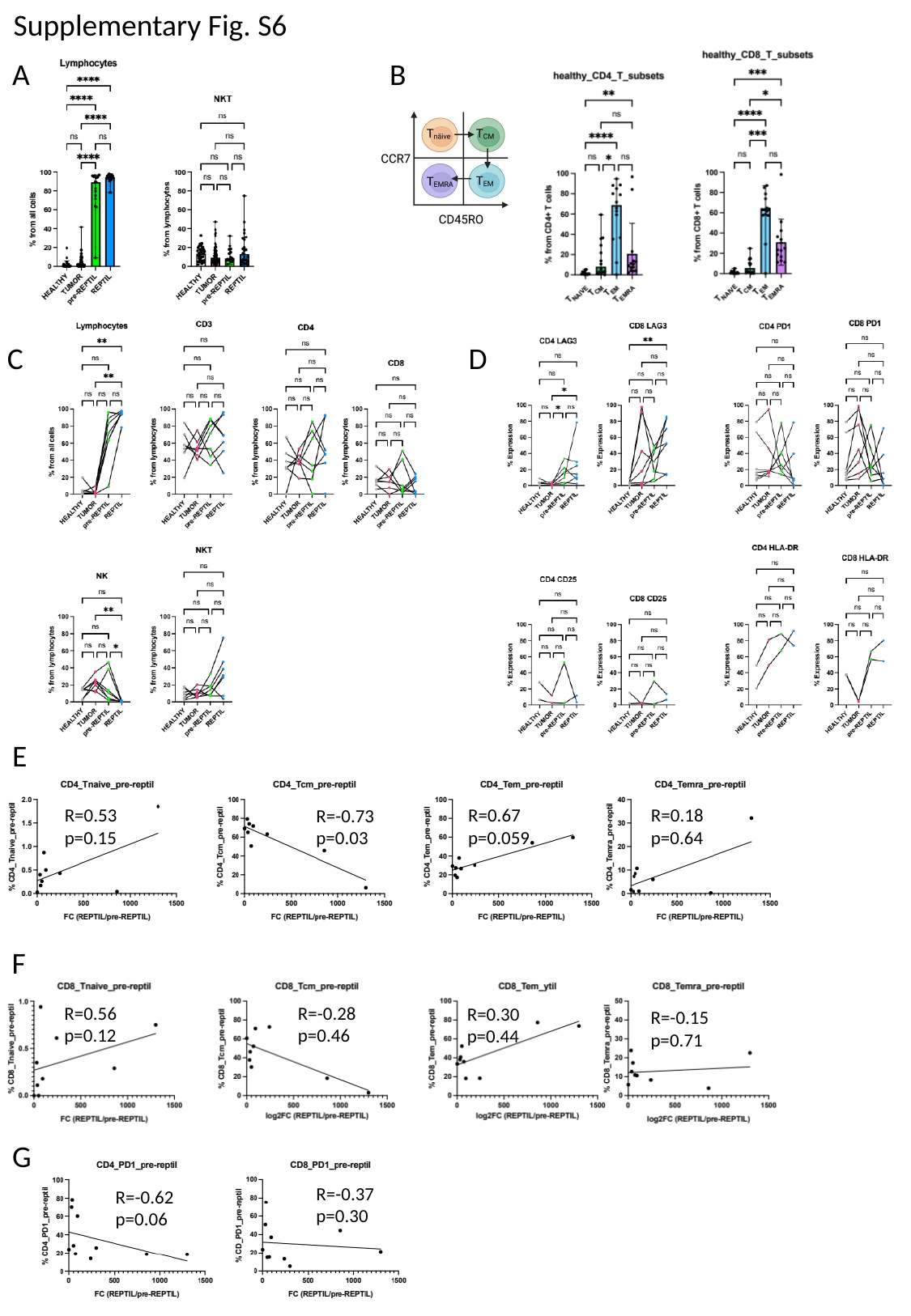

Supplementary Fig. S6
A
B
C
D
E
R=0.53
p=0.15
R=0.67
p=0.059
R=0.18
p=0.64
R=-0.73
p=0.03
F
R=-0.28
p=0.46
R=0.56
p=0.12
R=0.30
p=0.44
R=-0.15
p=0.71
G
R=-0.62
p=0.06
R=-0.37
p=0.30

## Slide 2
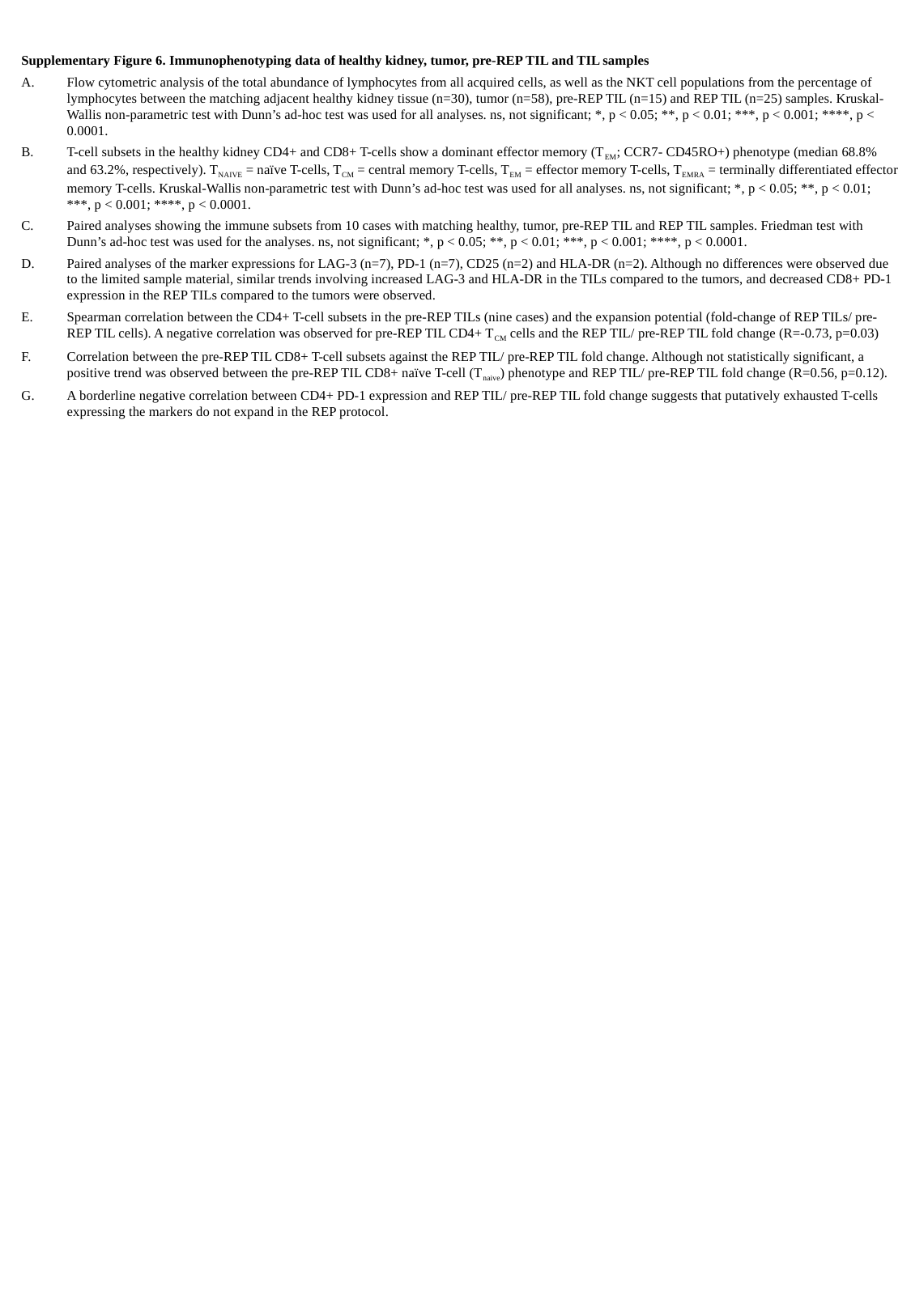

Supplementary Figure 6. Immunophenotyping data of healthy kidney, tumor, pre-REP TIL and TIL samples
Flow cytometric analysis of the total abundance of lymphocytes from all acquired cells, as well as the NKT cell populations from the percentage of lymphocytes between the matching adjacent healthy kidney tissue (n=30), tumor (n=58), pre-REP TIL (n=15) and REP TIL (n=25) samples. Kruskal-Wallis non-parametric test with Dunn’s ad-hoc test was used for all analyses. ns, not significant; *, p < 0.05; **, p < 0.01; ***, p < 0.001; ****, p < 0.0001.
T-cell subsets in the healthy kidney CD4+ and CD8+ T-cells show a dominant effector memory (TEM; CCR7- CD45RO+) phenotype (median 68.8% and 63.2%, respectively). TNAIVE = naïve T-cells, TCM = central memory T-cells, TEM = effector memory T-cells, TEMRA = terminally differentiated effector memory T-cells. Kruskal-Wallis non-parametric test with Dunn’s ad-hoc test was used for all analyses. ns, not significant; *, p < 0.05; **, p < 0.01; ***, p < 0.001; ****, p < 0.0001.
Paired analyses showing the immune subsets from 10 cases with matching healthy, tumor, pre-REP TIL and REP TIL samples. Friedman test with Dunn’s ad-hoc test was used for the analyses. ns, not significant; *, p < 0.05; **, p < 0.01; ***, p < 0.001; ****, p < 0.0001.
Paired analyses of the marker expressions for LAG-3 (n=7), PD-1 (n=7), CD25 (n=2) and HLA-DR (n=2). Although no differences were observed due to the limited sample material, similar trends involving increased LAG-3 and HLA-DR in the TILs compared to the tumors, and decreased CD8+ PD-1 expression in the REP TILs compared to the tumors were observed.
Spearman correlation between the CD4+ T-cell subsets in the pre-REP TILs (nine cases) and the expansion potential (fold-change of REP TILs/ pre-REP TIL cells). A negative correlation was observed for pre-REP TIL CD4+ TCM cells and the REP TIL/ pre-REP TIL fold change (R=-0.73, p=0.03)
Correlation between the pre-REP TIL CD8+ T-cell subsets against the REP TIL/ pre-REP TIL fold change. Although not statistically significant, a positive trend was observed between the pre-REP TIL CD8+ naïve T-cell (Tnaive) phenotype and REP TIL/ pre-REP TIL fold change (R=0.56, p=0.12).
A borderline negative correlation between CD4+ PD-1 expression and REP TIL/ pre-REP TIL fold change suggests that putatively exhausted T-cells expressing the markers do not expand in the REP protocol.
