# Supplementary material for: Immunologic Characterization and T cell Receptor Repertoires of Expanded Tumor-infiltrating Lymphocytes in Patients with Renal Cell Carcinoma
Source: Cancer Res Commun. 2023 Jul 18;3(7):1260–76. doi: 10.1158/2767-9764.CRC-22-0514 (PMC10361538; doi:10.1158/2767-9764.CRC-22-0514)
Supplement: Figure S7 — shows analyses of the co-culture assays involving CD107a/b, IFN-y,TNF-a and GzB expressions. [file crc-22-0514-s12.pptx]

## Slide 1
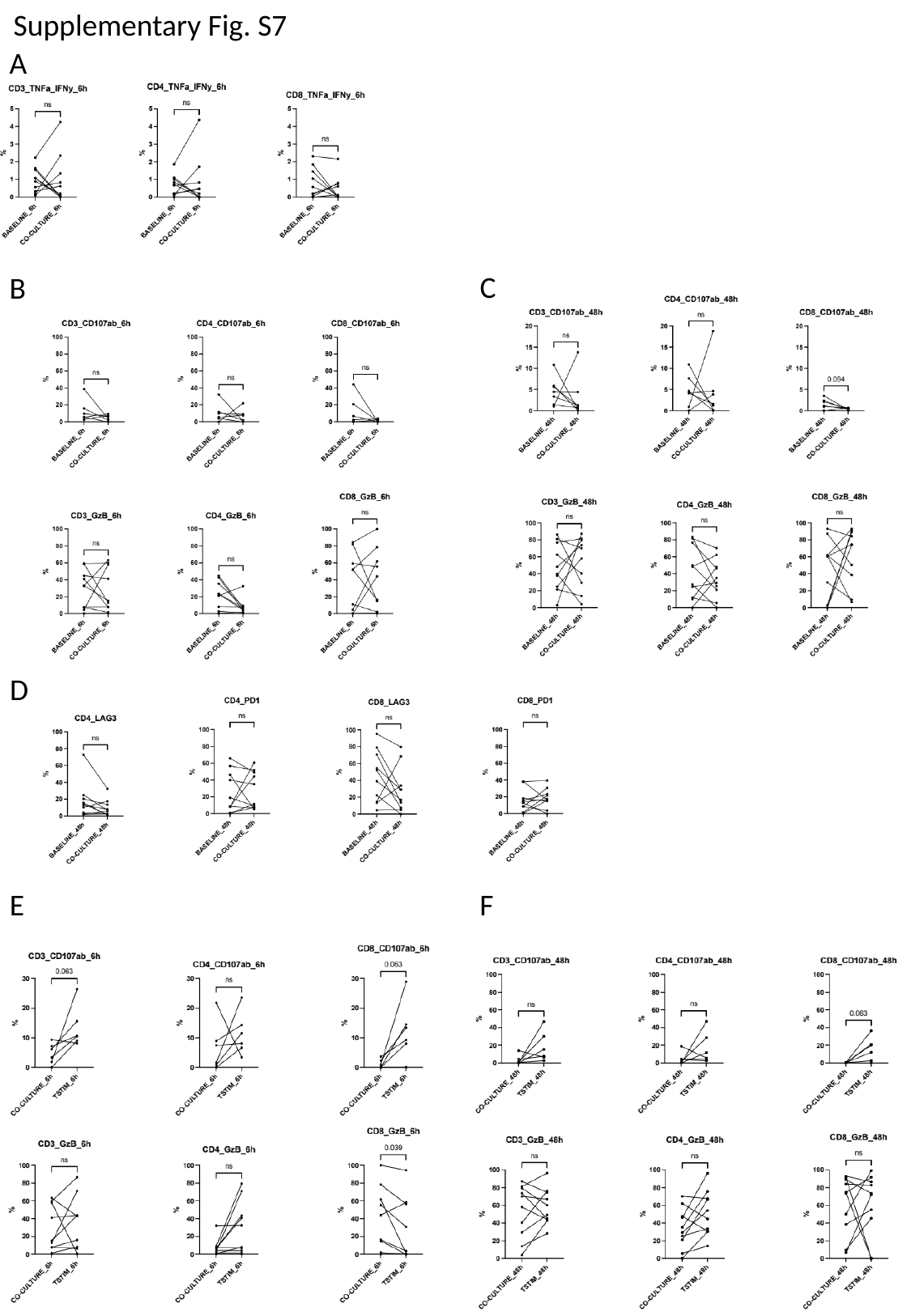

Supplementary Fig. S7
A
C
B
D
F
E

## Slide 2
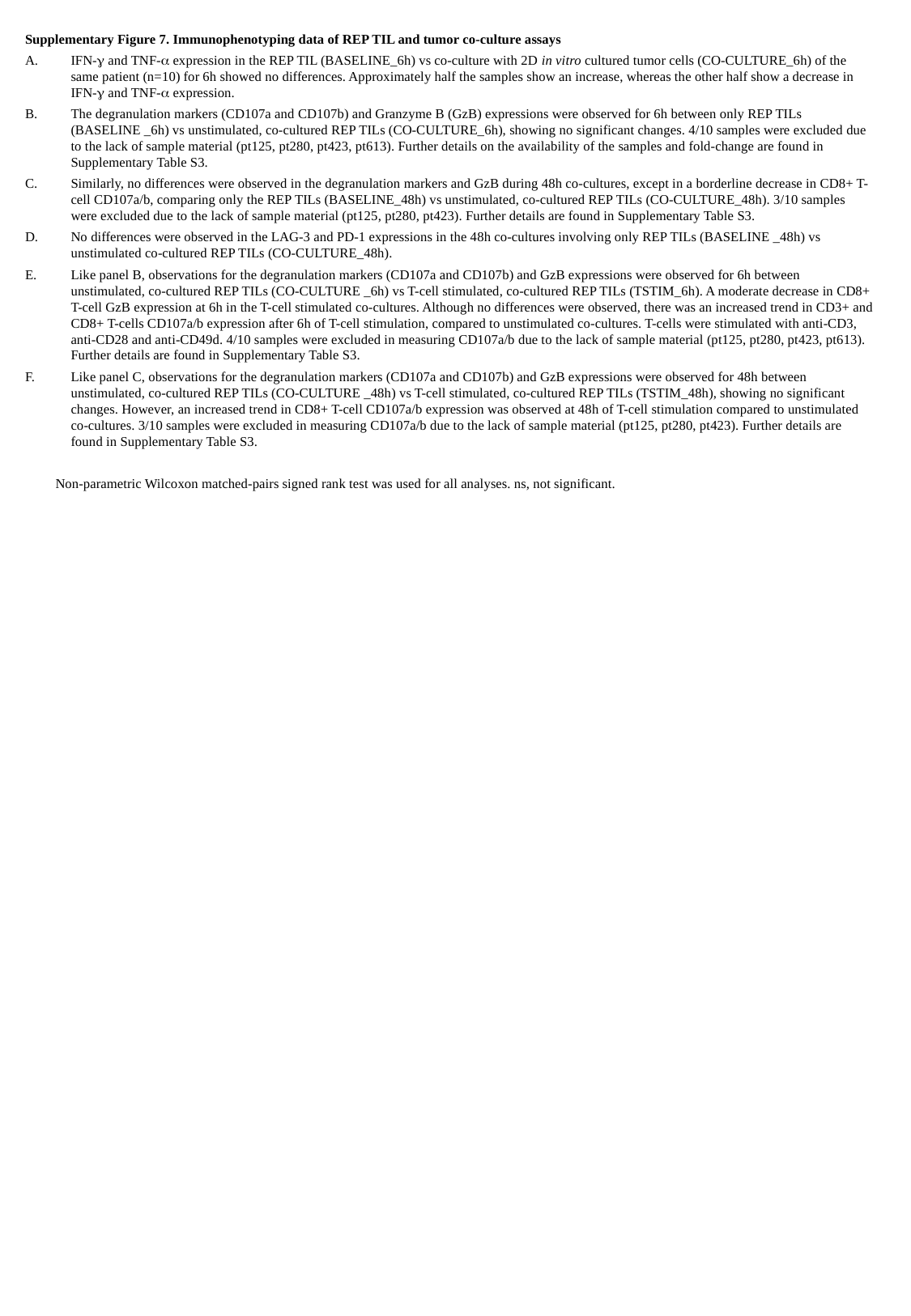

Supplementary Figure 7. Immunophenotyping data of REP TIL and tumor co-culture assays
IFN-γ and TNF-α expression in the REP TIL (BASELINE_6h) vs co-culture with 2D in vitro cultured tumor cells (CO-CULTURE_6h) of the same patient (n=10) for 6h showed no differences. Approximately half the samples show an increase, whereas the other half show a decrease in IFN-γ and TNF-α expression.
The degranulation markers (CD107a and CD107b) and Granzyme B (GzB) expressions were observed for 6h between only REP TILs (BASELINE _6h) vs unstimulated, co-cultured REP TILs (CO-CULTURE_6h), showing no significant changes. 4/10 samples were excluded due to the lack of sample material (pt125, pt280, pt423, pt613). Further details on the availability of the samples and fold-change are found in Supplementary Table S3.
Similarly, no differences were observed in the degranulation markers and GzB during 48h co-cultures, except in a borderline decrease in CD8+ T-cell CD107a/b, comparing only the REP TILs (BASELINE_48h) vs unstimulated, co-cultured REP TILs (CO-CULTURE_48h). 3/10 samples were excluded due to the lack of sample material (pt125, pt280, pt423). Further details are found in Supplementary Table S3.
No differences were observed in the LAG-3 and PD-1 expressions in the 48h co-cultures involving only REP TILs (BASELINE _48h) vs unstimulated co-cultured REP TILs (CO-CULTURE_48h).
Like panel B, observations for the degranulation markers (CD107a and CD107b) and GzB expressions were observed for 6h between unstimulated, co-cultured REP TILs (CO-CULTURE _6h) vs T-cell stimulated, co-cultured REP TILs (TSTIM_6h). A moderate decrease in CD8+ T-cell GzB expression at 6h in the T-cell stimulated co-cultures. Although no differences were observed, there was an increased trend in CD3+ and CD8+ T-cells CD107a/b expression after 6h of T-cell stimulation, compared to unstimulated co-cultures. T-cells were stimulated with anti-CD3, anti-CD28 and anti-CD49d. 4/10 samples were excluded in measuring CD107a/b due to the lack of sample material (pt125, pt280, pt423, pt613). Further details are found in Supplementary Table S3.
Like panel C, observations for the degranulation markers (CD107a and CD107b) and GzB expressions were observed for 48h between unstimulated, co-cultured REP TILs (CO-CULTURE _48h) vs T-cell stimulated, co-cultured REP TILs (TSTIM_48h), showing no significant changes. However, an increased trend in CD8+ T-cell CD107a/b expression was observed at 48h of T-cell stimulation compared to unstimulated co-cultures. 3/10 samples were excluded in measuring CD107a/b due to the lack of sample material (pt125, pt280, pt423). Further details are found in Supplementary Table S3.
Non-parametric Wilcoxon matched-pairs signed rank test was used for all analyses. ns, not significant.
