# Supplementary material for: Immunologic Characterization and T cell Receptor Repertoires of Expanded Tumor-infiltrating Lymphocytes in Patients with Renal Cell Carcinoma
Source: Cancer Res Commun. 2023 Jul 18;3(7):1260–76. doi: 10.1158/2767-9764.CRC-22-0514 (PMC10361538; doi:10.1158/2767-9764.CRC-22-0514)
Supplement: Figure S8 — shows the analyses of the different clonotype sizes and tracking their abundancies found in the bulk TCRb-seq data. [file crc-22-0514-s13.pptx]

## Slide 1
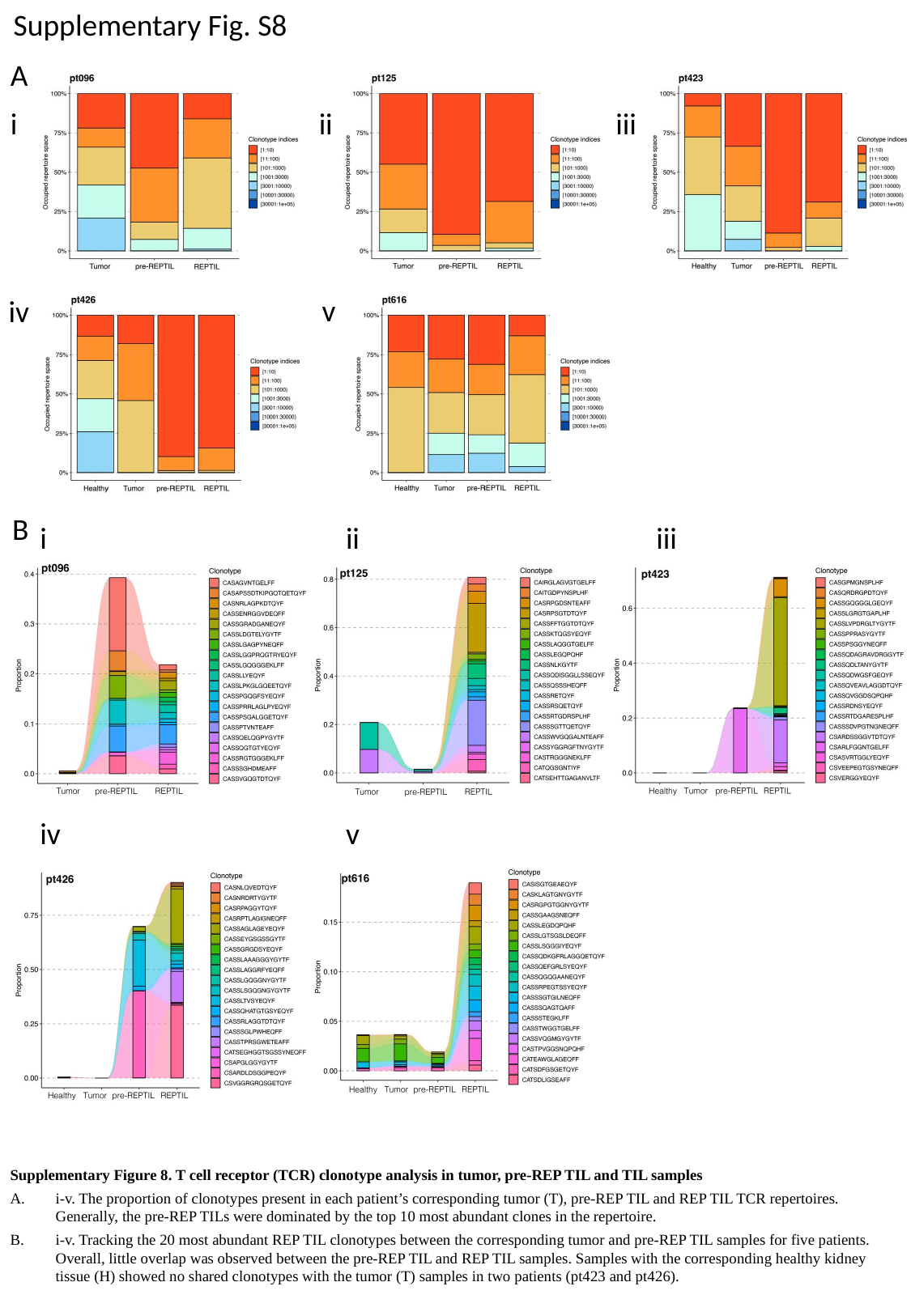

Supplementary Fig. S8
A
i
ii
iii
v
iv
B
i
ii
iii
iv
v
Supplementary Figure 8. T cell receptor (TCR) clonotype analysis in tumor, pre-REP TIL and TIL samples
i-v. The proportion of clonotypes present in each patient’s corresponding tumor (T), pre-REP TIL and REP TIL TCR repertoires. Generally, the pre-REP TILs were dominated by the top 10 most abundant clones in the repertoire.
i-v. Tracking the 20 most abundant REP TIL clonotypes between the corresponding tumor and pre-REP TIL samples for five patients. Overall, little overlap was observed between the pre-REP TIL and REP TIL samples. Samples with the corresponding healthy kidney tissue (H) showed no shared clonotypes with the tumor (T) samples in two patients (pt423 and pt426).
