# Supplementary material for: Immunologic Characterization and T cell Receptor Repertoires of Expanded Tumor-infiltrating Lymphocytes in Patients with Renal Cell Carcinoma
Source: Cancer Res Commun. 2023 Jul 18;3(7):1260–76. doi: 10.1158/2767-9764.CRC-22-0514 (PMC10361538; doi:10.1158/2767-9764.CRC-22-0514)
Supplement: Figure S9 — shows the correlation of overlapping clonotypes between the REP TILs and pre-REP TILs. [file crc-22-0514-s14.pptx]

## Slide 1
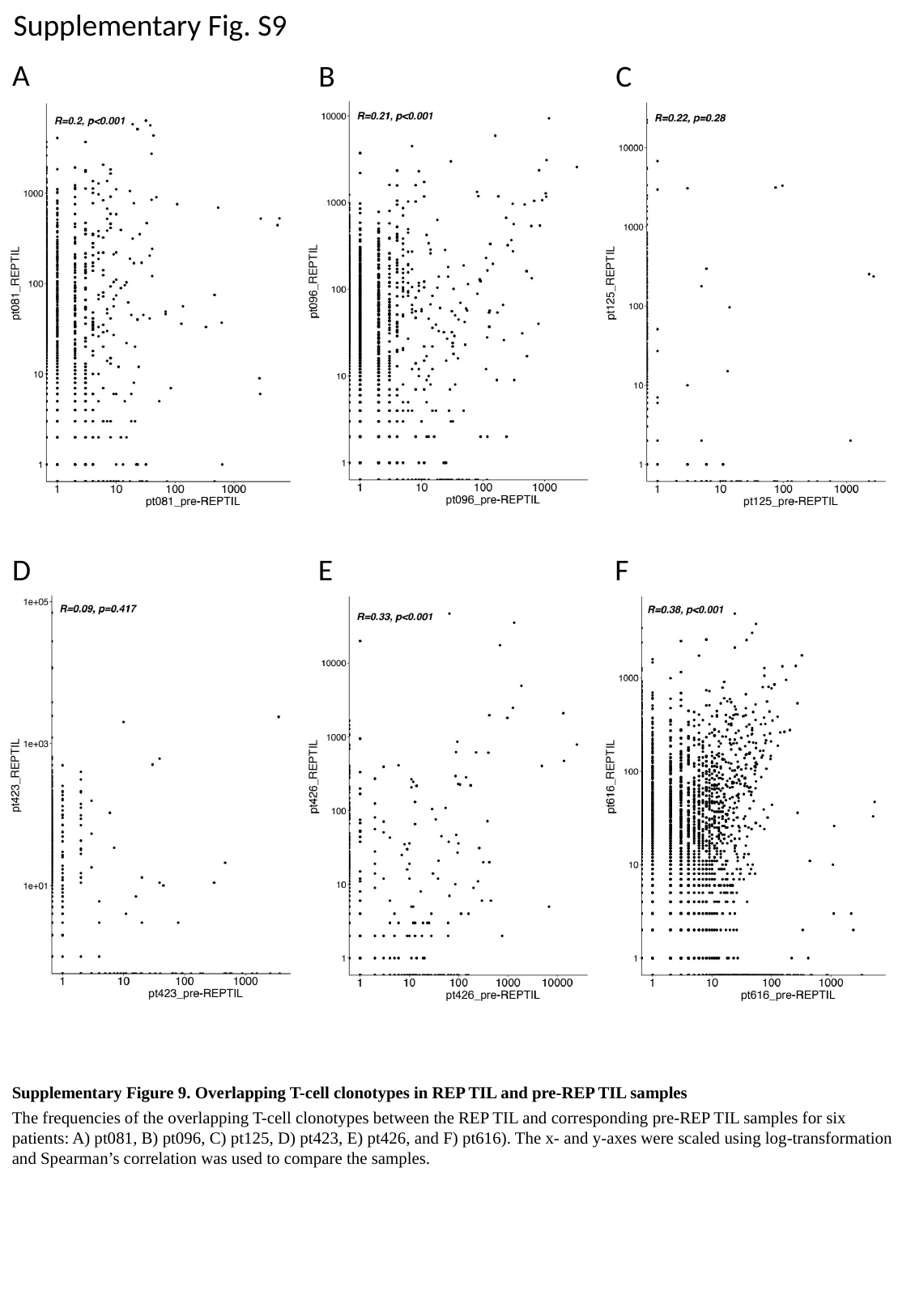

Supplementary Fig. S9
A
B
C
D
E
F
Supplementary Figure 9. Overlapping T-cell clonotypes in REP TIL and pre-REP TIL samples
The frequencies of the overlapping T-cell clonotypes between the REP TIL and corresponding pre-REP TIL samples for six patients: A) pt081, B) pt096, C) pt125, D) pt423, E) pt426, and F) pt616). The x- and y-axes were scaled using log-transformation and Spearman’s correlation was used to compare the samples.
