# Supplementary material for: Immunologic Characterization and T cell Receptor Repertoires of Expanded Tumor-infiltrating Lymphocytes in Patients with Renal Cell Carcinoma
Source: Cancer Res Commun. 2023 Jul 18;3(7):1260–76. doi: 10.1158/2767-9764.CRC-22-0514 (PMC10361538; doi:10.1158/2767-9764.CRC-22-0514)
Supplement: Figure S11 — shows treemaps of various clonotypes found in the patient samples and the matches to viral-specific TCRs. [file crc-22-0514-s16.pptx]

## Slide 1
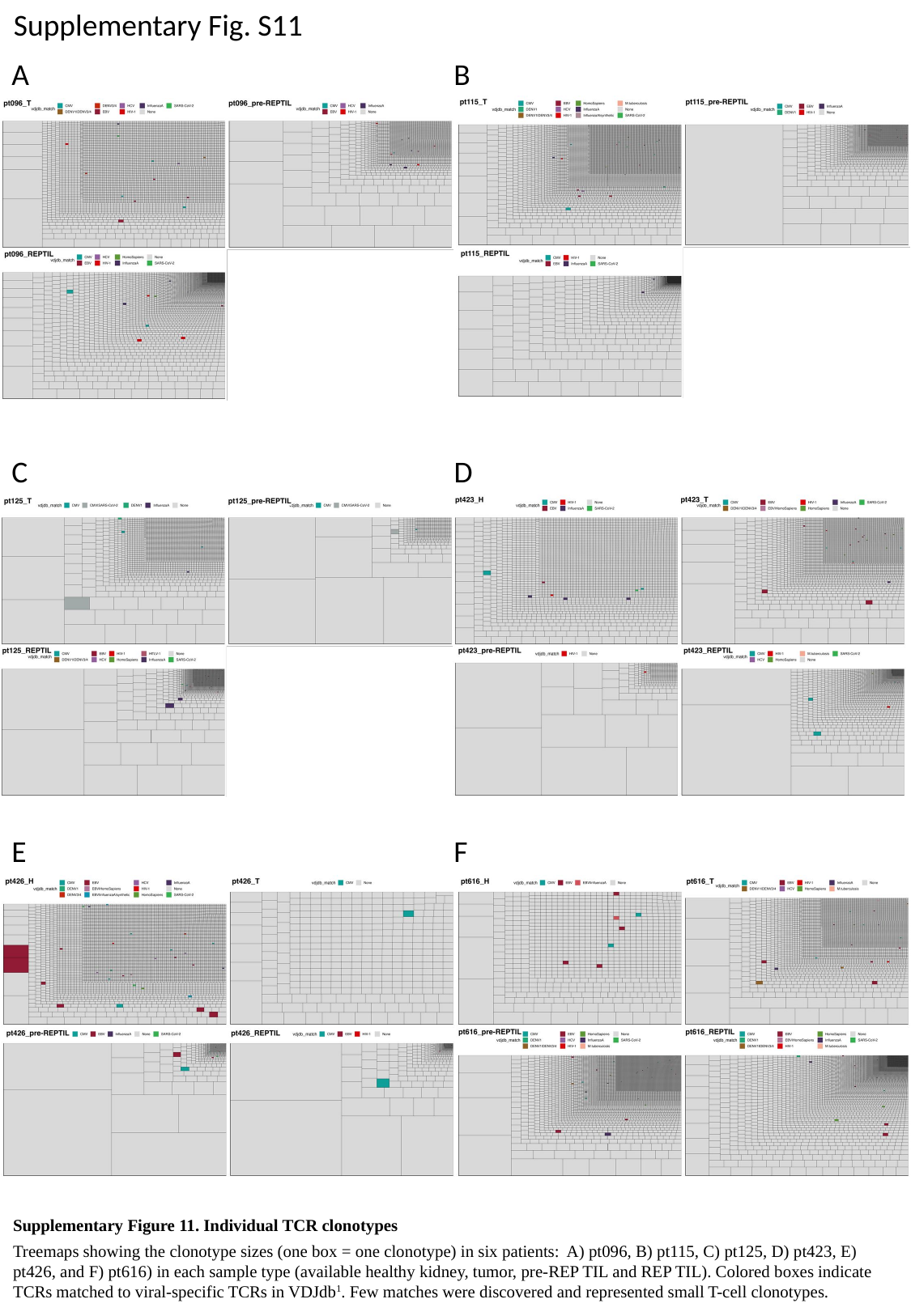

Supplementary Fig. S11
A
B
C
D
E
F
Supplementary Figure 11. Individual TCR clonotypes
Treemaps showing the clonotype sizes (one box = one clonotype) in six patients: A) pt096, B) pt115, C) pt125, D) pt423, E) pt426, and F) pt616) in each sample type (available healthy kidney, tumor, pre-REP TIL and REP TIL). Colored boxes indicate TCRs matched to viral-specific TCRs in VDJdb1. Few matches were discovered and represented small T-cell clonotypes.
