# Supplementary material for: Immunologic Characterization and T cell Receptor Repertoires of Expanded Tumor-infiltrating Lymphocytes in Patients with Renal Cell Carcinoma
Source: Cancer Res Commun. 2023 Jul 18;3(7):1260–76. doi: 10.1158/2767-9764.CRC-22-0514 (PMC10361538; doi:10.1158/2767-9764.CRC-22-0514)
Supplement: Figure S12 — shows the validation of RCC-associated TCR motifs in various cohorts. [file crc-22-0514-s17.pptx]

## Slide 1
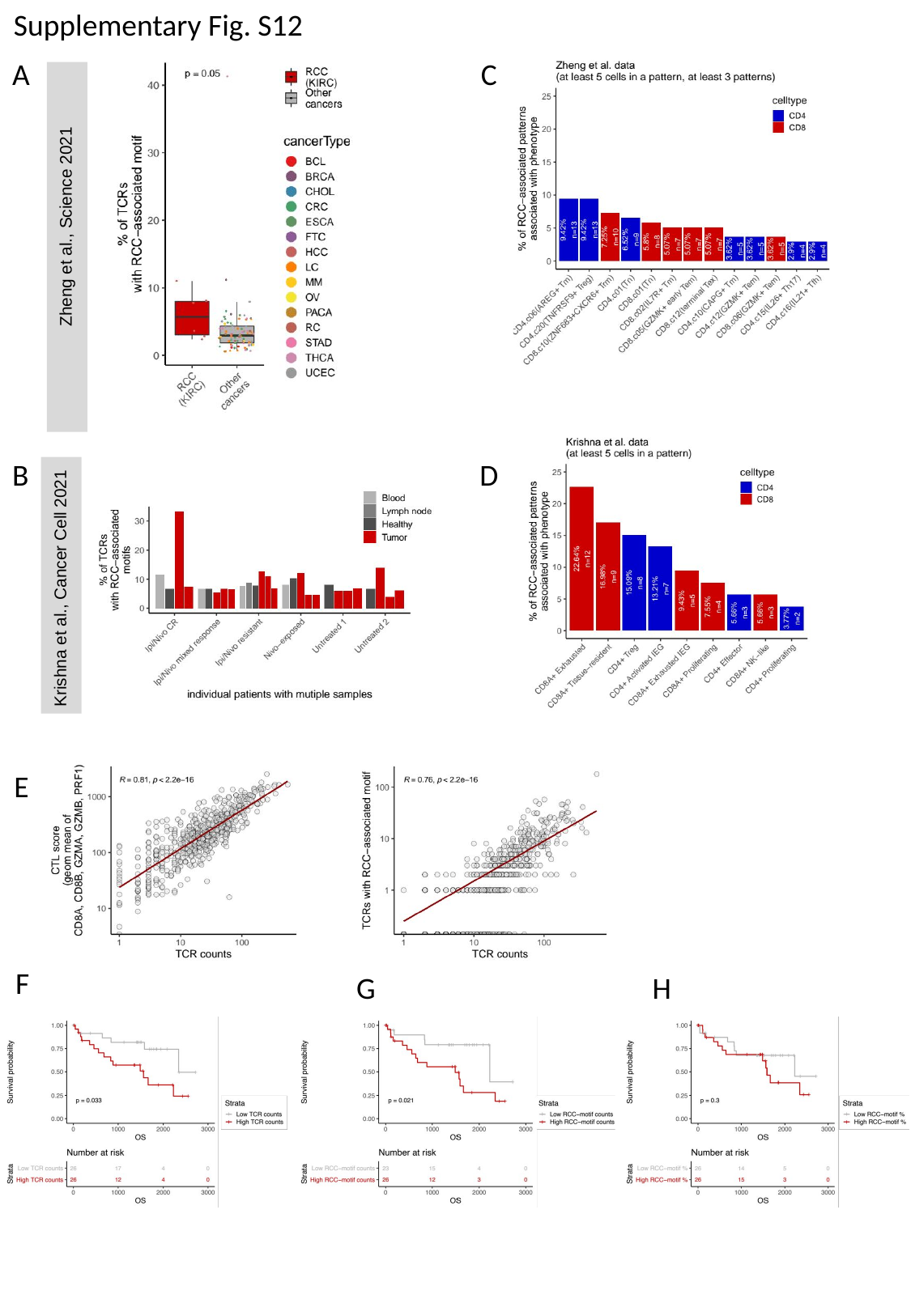

Supplementary Fig. S12
A
C
Zheng et al., Science 2021
B
D
Krishna et al., Cancer Cell 2021
E
F
G
H

## Slide 2
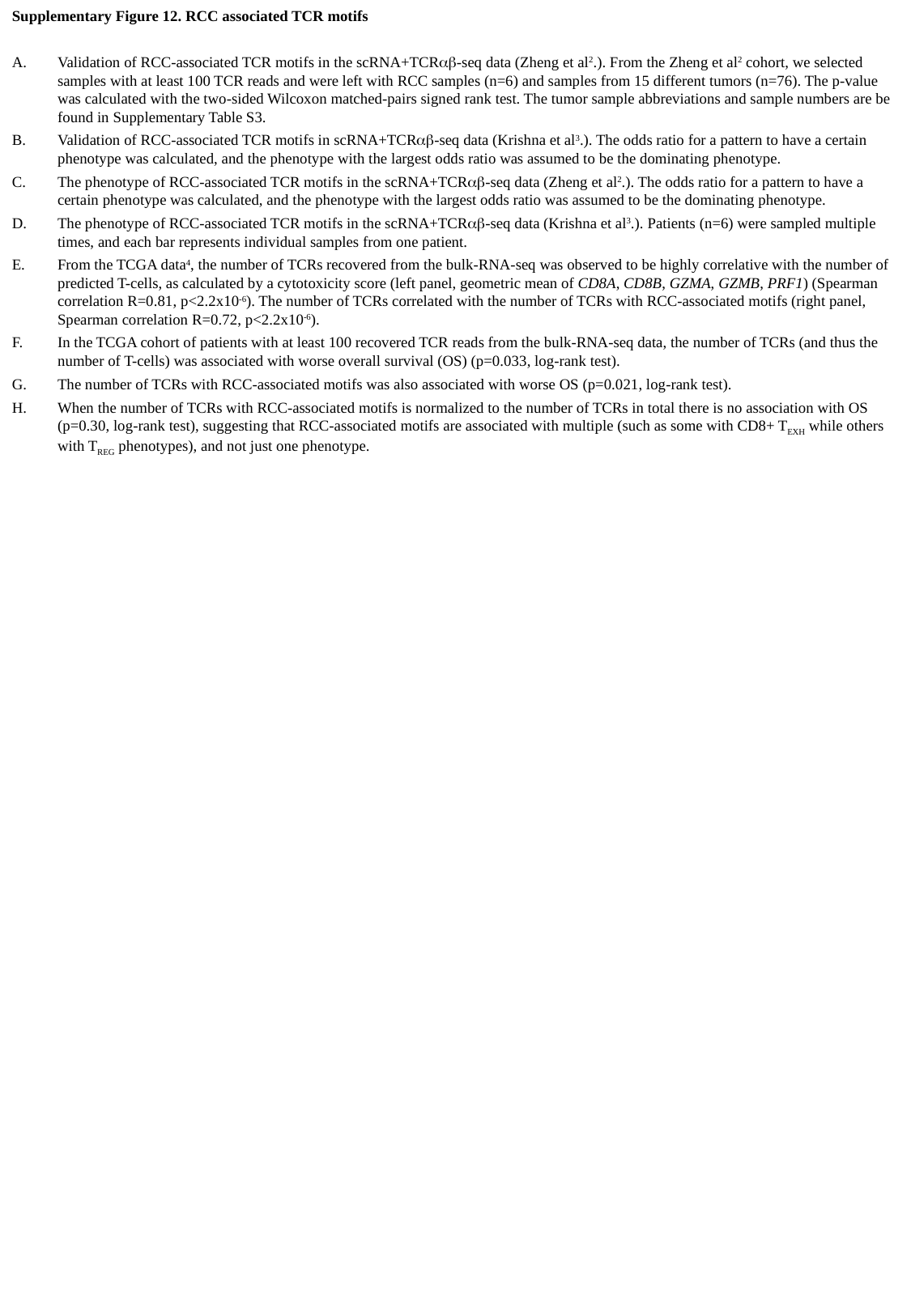

Supplementary Figure 12. RCC associated TCR motifs
Validation of RCC-associated TCR motifs in the scRNA+TCRαβ-seq data (Zheng et al2.). From the Zheng et al2 cohort, we selected samples with at least 100 TCR reads and were left with RCC samples (n=6) and samples from 15 different tumors (n=76). The p-value was calculated with the two-sided Wilcoxon matched-pairs signed rank test. The tumor sample abbreviations and sample numbers are be found in Supplementary Table S3.
Validation of RCC-associated TCR motifs in scRNA+TCRαβ-seq data (Krishna et al3.). The odds ratio for a pattern to have a certain phenotype was calculated, and the phenotype with the largest odds ratio was assumed to be the dominating phenotype.
The phenotype of RCC-associated TCR motifs in the scRNA+TCRαβ-seq data (Zheng et al2.). The odds ratio for a pattern to have a certain phenotype was calculated, and the phenotype with the largest odds ratio was assumed to be the dominating phenotype.
The phenotype of RCC-associated TCR motifs in the scRNA+TCRαβ-seq data (Krishna et al3.). Patients (n=6) were sampled multiple times, and each bar represents individual samples from one patient.
From the TCGA data4, the number of TCRs recovered from the bulk-RNA-seq was observed to be highly correlative with the number of predicted T-cells, as calculated by a cytotoxicity score (left panel, geometric mean of CD8A, CD8B, GZMA, GZMB, PRF1) (Spearman correlation R=0.81, p<2.2x10^-6^). The number of TCRs correlated with the number of TCRs with RCC-associated motifs (right panel, Spearman correlation R=0.72, p<2.2x10^-6^).
In the TCGA cohort of patients with at least 100 recovered TCR reads from the bulk-RNA-seq data, the number of TCRs (and thus the number of T-cells) was associated with worse overall survival (OS) (p=0.033, log-rank test).
The number of TCRs with RCC-associated motifs was also associated with worse OS (p=0.021, log-rank test).
When the number of TCRs with RCC-associated motifs is normalized to the number of TCRs in total there is no association with OS (p=0.30, log-rank test), suggesting that RCC-associated motifs are associated with multiple (such as some with CD8+ TEXH while others with TREG phenotypes), and not just one phenotype.
