# Supplementary material for: Immunologic Characterization and T cell Receptor Repertoires of Expanded Tumor-infiltrating Lymphocytes in Patients with Renal Cell Carcinoma
Source: Cancer Res Commun. 2023 Jul 18;3(7):1260–76. doi: 10.1158/2767-9764.CRC-22-0514 (PMC10361538; doi:10.1158/2767-9764.CRC-22-0514)
Supplement: Figure S13 — shows analyses from the scRNA+TCRab-seq data, including the different UMAP clusters, differentially expressed genes and canonical marker gene expressions. [file crc-22-0514-s18.pptx]

## Slide 1
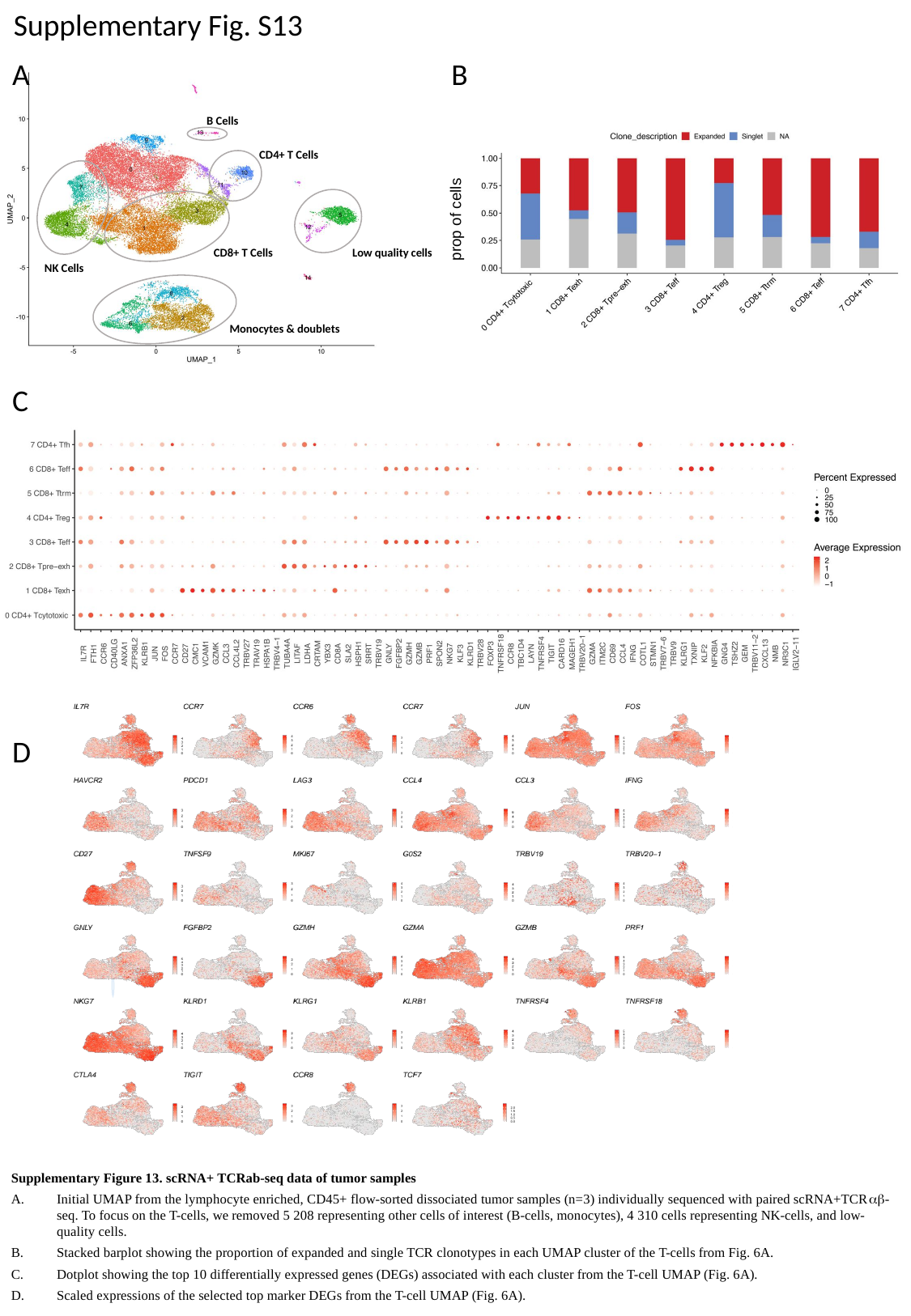

Supplementary Fig. S13
A
B
B Cells
CD4+ T Cells
CD8+ T Cells
Low quality cells
NK Cells
Monocytes & doublets
prop of cells
C
D
Supplementary Figure 13. scRNA+ TCRab-seq data of tumor samples
Initial UMAP from the lymphocyte enriched, CD45+ flow-sorted dissociated tumor samples (n=3) individually sequenced with paired scRNA+TCRαβ-seq. To focus on the T-cells, we removed 5 208 representing other cells of interest (B-cells, monocytes), 4 310 cells representing NK-cells, and low-quality cells.
Stacked barplot showing the proportion of expanded and single TCR clonotypes in each UMAP cluster of the T-cells from Fig. 6A.
Dotplot showing the top 10 differentially expressed genes (DEGs) associated with each cluster from the T-cell UMAP (Fig. 6A).
Scaled expressions of the selected top marker DEGs from the T-cell UMAP (Fig. 6A).
