# Supplementary material for: Immunologic Characterization and T cell Receptor Repertoires of Expanded Tumor-infiltrating Lymphocytes in Patients with Renal Cell Carcinoma
Source: Cancer Res Commun. 2023 Jul 18;3(7):1260–76. doi: 10.1158/2767-9764.CRC-22-0514 (PMC10361538; doi:10.1158/2767-9764.CRC-22-0514)
Supplement: Figure S14 — shows the clonalities, expression scores and size of clonotypes represented in the T-cell UMAP clusters. [file crc-22-0514-s19.pptx]

## Slide 1
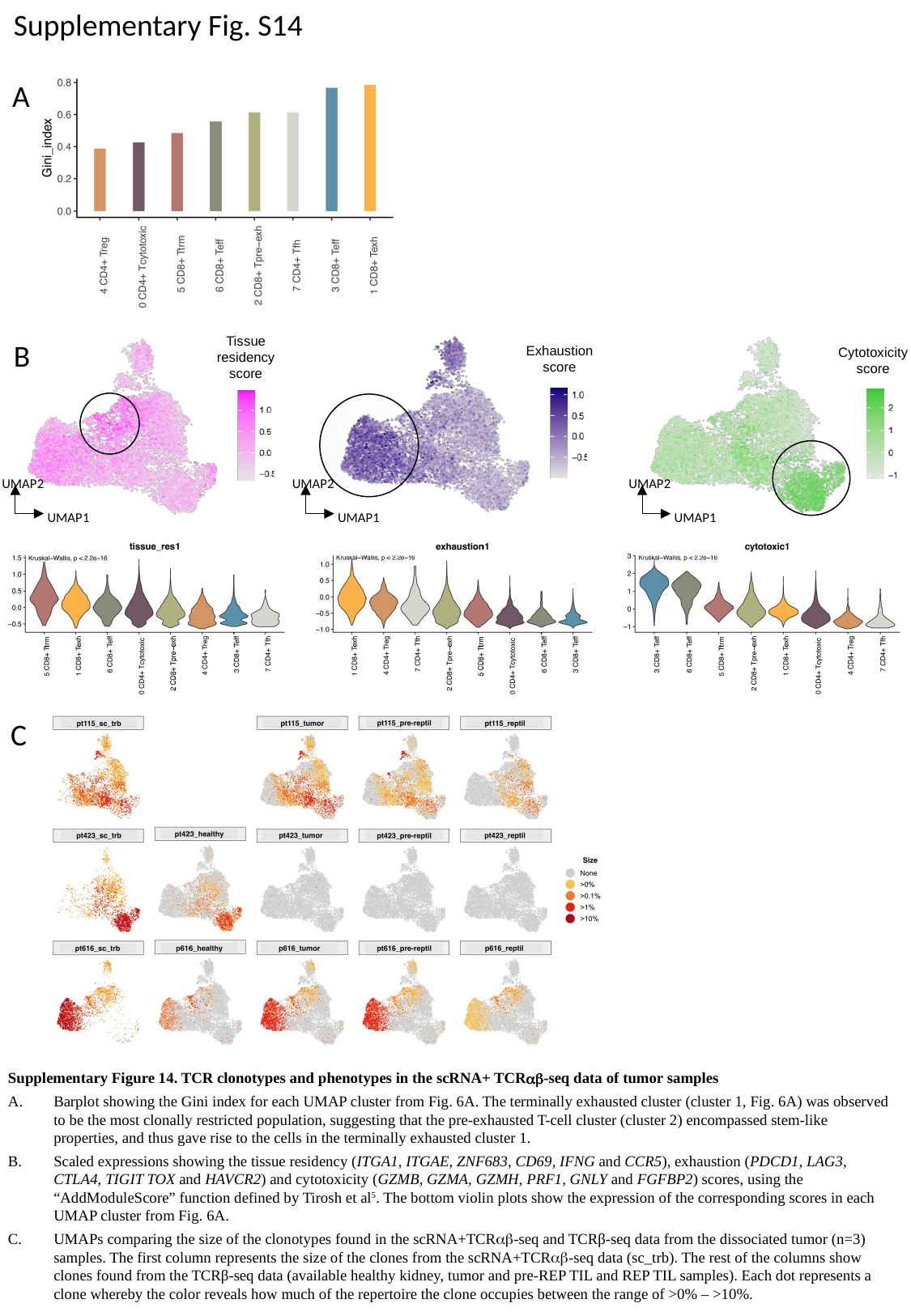

Supplementary Fig. S14
A
Tissue residency score
UMAP2
UMAP1
B
Cytotoxicity score
UMAP2
UMAP1
Exhaustion score
UMAP2
UMAP1
C
Supplementary Figure 14. TCR clonotypes and phenotypes in the scRNA+ TCRαβ-seq data of tumor samples
Barplot showing the Gini index for each UMAP cluster from Fig. 6A. The terminally exhausted cluster (cluster 1, Fig. 6A) was observed to be the most clonally restricted population, suggesting that the pre-exhausted T-cell cluster (cluster 2) encompassed stem-like properties, and thus gave rise to the cells in the terminally exhausted cluster 1.
Scaled expressions showing the tissue residency (ITGA1, ITGAE, ZNF683, CD69, IFNG and CCR5), exhaustion (PDCD1, LAG3, CTLA4, TIGIT TOX and HAVCR2) and cytotoxicity (GZMB, GZMA, GZMH, PRF1, GNLY and FGFBP2) scores, using the “AddModuleScore” function defined by Tirosh et al5. The bottom violin plots show the expression of the corresponding scores in each UMAP cluster from Fig. 6A.
UMAPs comparing the size of the clonotypes found in the scRNA+TCRαβ-seq and TCRβ-seq data from the dissociated tumor (n=3) samples. The first column represents the size of the clones from the scRNA+TCRαβ-seq data (sc_trb). The rest of the columns show clones found from the TCRβ-seq data (available healthy kidney, tumor and pre-REP TIL and REP TIL samples). Each dot represents a clone whereby the color reveals how much of the repertoire the clone occupies between the range of >0% – >10%.
