# Supplementary material for: Immunologic Characterization and T cell Receptor Repertoires of Expanded Tumor-infiltrating Lymphocytes in Patients with Renal Cell Carcinoma
Source: Cancer Res Commun. 2023 Jul 18;3(7):1260–76. doi: 10.1158/2767-9764.CRC-22-0514 (PMC10361538; doi:10.1158/2767-9764.CRC-22-0514)
Supplement: Figure S15 — shows pathway enrichment analyses for the pre-REP TILs and REP TILs, as well as the abundance of the RCC-associated motifs found in the UMAP T-cell clusters for each tumor sample. [file crc-22-0514-s20.pptx]

## Slide 1
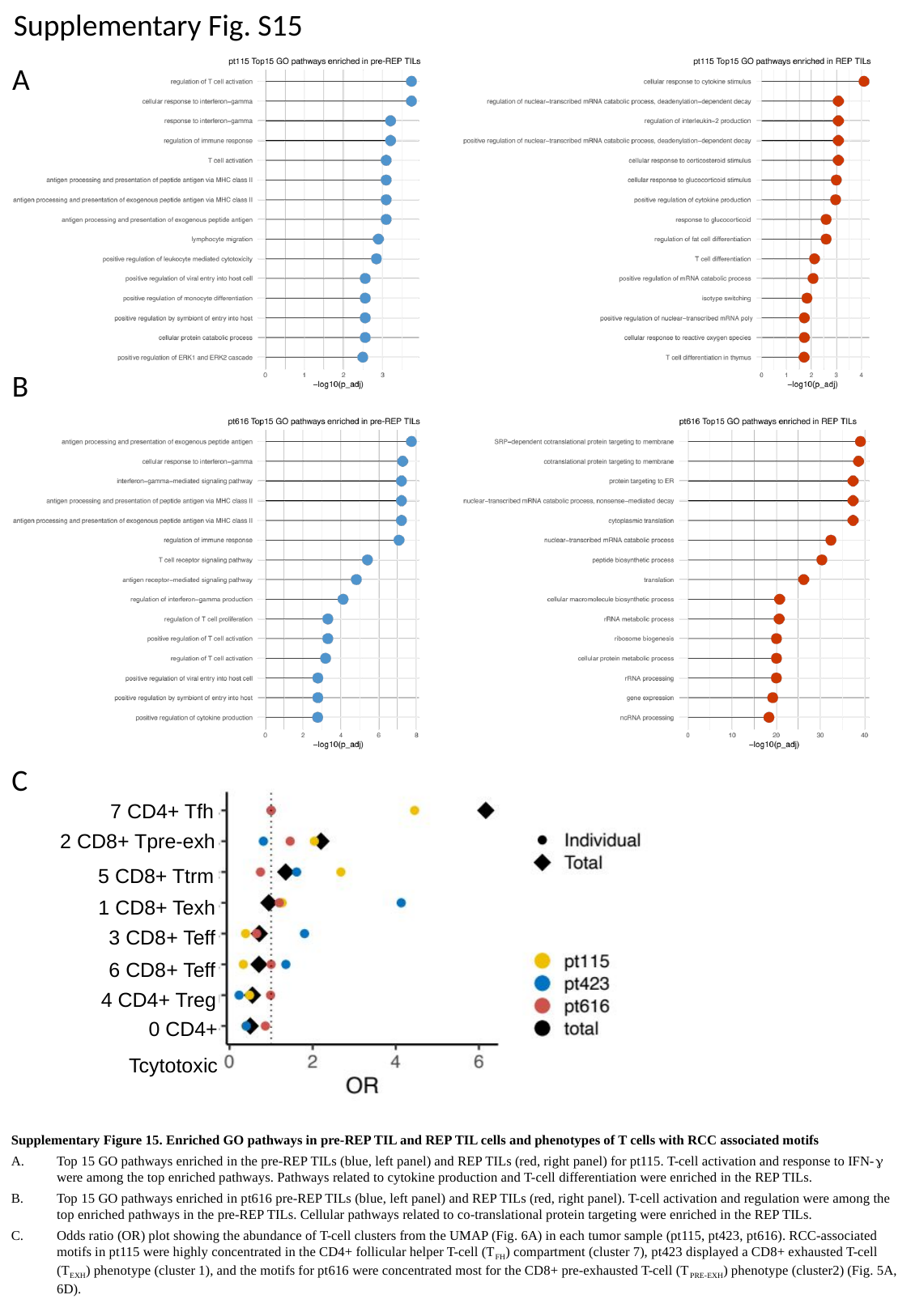

Supplementary Fig. S15
A
B
C
7 CD4+ Tfh
2 CD8+ Tpre-exh
5 CD8+ Ttrm
1 CD8+ Texh
3 CD8+ Teff
6 CD8+ Teff
4 CD4+ Treg
0 CD4+ Tcytotoxic
Supplementary Figure 15. Enriched GO pathways in pre-REP TIL and REP TIL cells and phenotypes of T cells with RCC associated motifs
Top 15 GO pathways enriched in the pre-REP TILs (blue, left panel) and REP TILs (red, right panel) for pt115. T-cell activation and response to IFN-γ were among the top enriched pathways. Pathways related to cytokine production and T-cell differentiation were enriched in the REP TILs.
Top 15 GO pathways enriched in pt616 pre-REP TILs (blue, left panel) and REP TILs (red, right panel). T-cell activation and regulation were among the top enriched pathways in the pre-REP TILs. Cellular pathways related to co-translational protein targeting were enriched in the REP TILs.
Odds ratio (OR) plot showing the abundance of T-cell clusters from the UMAP (Fig. 6A) in each tumor sample (pt115, pt423, pt616). RCC-associated motifs in pt115 were highly concentrated in the CD4+ follicular helper T-cell (TFH) compartment (cluster 7), pt423 displayed a CD8+ exhausted T-cell (TEXH) phenotype (cluster 1), and the motifs for pt616 were concentrated most for the CD8+ pre-exhausted T-cell (TPRE-EXH) phenotype (cluster2) (Fig. 5A, 6D).
